# In-depth Mendelian randomization analysis of causal factors for coronary artery disease

**DOI:** 10.1038/s41598-020-66027-4

**Published:** 2020-06-08

**Authors:** Yuan-De Tan, Peng Xiao, Chittibabu Guda

**Affiliations:** Department of Genetics, Cell Biology & Anatomy in Nebraska University Medical Center, Omaha, NE 68198 USA

**Keywords:** Statistical methods, Genome-wide association studies

## Abstract

Selecting a set of valid genetic variants is critical for Mendelian randomization (MR) to correctly infer risk factors causing a disease. We here developed a method for selecting genetic variants as valid instrumental variables for inferring risk factors causing coronary artery disease (CAD). Using this method, we selected two sets of single-nucleotide-polymorphism (SNP) genetic variants (SNP338 and SNP363) associated with each of the three potential risk factors for CAD including low density lipoprotein cholesterol (LDL-c), high density lipoprotein cholesterol (HDL-c) and triglycerides (TG) from two independent GWAS datasets. We performed in-depth multivariate MR (MVMR) analyses and the results from both datasets consistently showed that LDL-c was strongly associated with increased risk for CAD (*β* = 0.396,OR = 1.486 per 1 SD (equivalent to 38 mg/dL), 95CI = (1.38, 1.59) in SNP338; and *β* = 0.424, OR = 1.528 per 1 SD, 95%CI = (1.42, 1.65) in SNP363); HDL-c was strongly associated with reduced risk for CAD (*β* = −0.315, OR = 0.729 per 1 SD (equivalent to 16 mg/dL), 95CI = (0.68, 0.78) in SNP338; and *β* = −0.319, OR = 0.726 per 1 SD, 95%CI = (0.66, 0.80), in SNP363). In case of TG, when using the full datasets, an increased risk for CAD (*β* = 0.184, OR = 1.2 per 1 SD (equivalent to 89 mg/dL), 95%CI = (1.12, 1.28) in SNPP338; and *β* = 0.207, OR = 1.222 per 1 SD, 95%CI = (1.10, 1.36) in SNP363) was observed, while using partial datasets that contain shared and unique SNPs showed that TG is not a risk factor for CAD. From these results, it can be inferred that TG itself is not a causal risk factor for CAD, but it’s shown as a risk factor due to pleiotropic effects associated with LDL-c and HDL-c SNPs. Large-scale simulation experiments without pleiotropic effects also corroborated these results.

## Introduction

Coronary artery disease (CAD) is one of the leading causes of morbidity and mortality worldwide^1^. Association of the risks of lipoprotein cholesterols with risk for CAD has been extensively investigated to date. Causal influence of high levels of low-density lipoprotein cholesterol (LDL-c) in blood on CAD has been generally accepted^2–7^. However, the roles of high-density lipoprotein cholesterol (HDL-c) and triglycerides (TG) in causing CAD have still been unclear and controversial^8–16^.

**Figure 1:**
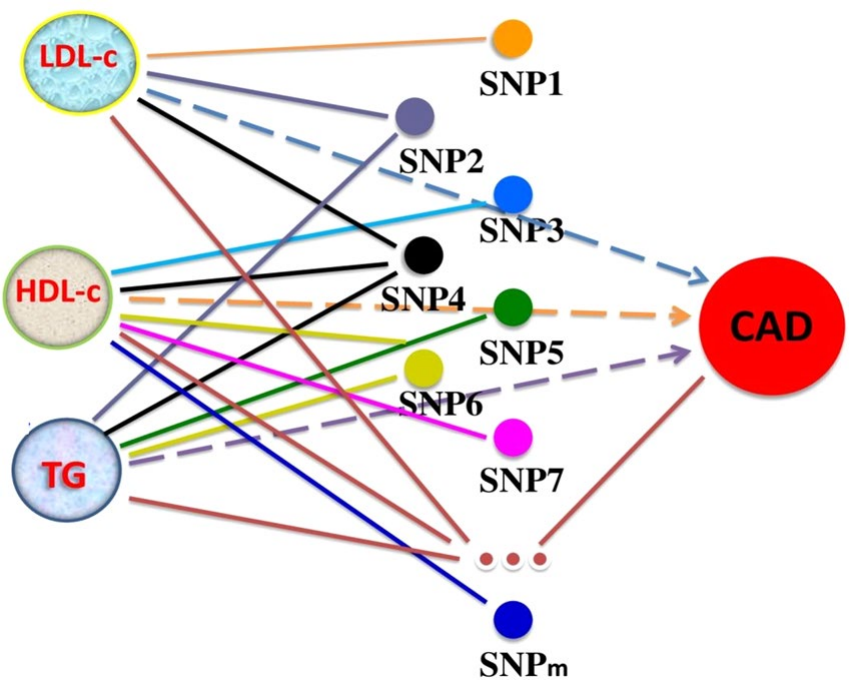
Schematic showing the principle of multiple Mendelian randomization analyses. Multiple SNPs were selected from GWAS data as instrumental variables for Mendelian randomization (MR) analyses to identify multiple risk factors LDL-c, HDL-c and TG that may cause CAD. Lines without arrow denote association between two objects. A dash line with arrow denotes an undefined causality. These SNPs are associated with at least one of the three risk factors. SNPs that are associated with two or more risk factors have pleiotropic effects.

Mendelian randomization (MR) is an effective approach to evaluate causal relationship between a biological factor (i.e., single variable) and a disease of interest^17,18^. Many investigators have attempted to use MR to demonstrate which lipoprotein cholesterols are risk factors for CAD^14,15,19–33^. However, when multiple factors are involved, MR analysis becomes complicated^17,34^ due to pleiotropy^35^ or interactions between factors. Moreover, a single genetic variant may provide a biased or false instrument for inferring causality of the disease^3,10,15,36^ because most biological risk factors are controlled by multiple genes. This issue may be addressed by using a set of valid genetic variants as instrumental variables^37^ (Fig. 1). However, how many genetic variants are required for an effective instrument and which genetic variants are valid instrumental variables should be addressed in the MR analysis. These two issues may be attributed to one issue: how do we select a set of valid genetic variants? This is the basis for accurate MR inference of causal factors for a disease. Recently Do *et al*.^3^ reported an empirical method to select 185 single nucleotide polymorphisms (SNPs) from GWAS consortium meta-analysis data.

Using these 185 SNPs as instrumental variables, they found out that LDL-c and TG are factors that increase the risk for CAD, but HDL-c was not associated with risk for CAD. Using the same SNP dataset, Bowden *et al*.^20,38^ and White *et al*.^15^ obtained similar results. However, we found that SNP selection of Do *et al*.^3^ is not reproducible and no evidence shows that these 185 SNPs are ideal for their use as valid multiple instrumental variables for MR to infer which lipoprotein cholesterols are risk factors causing CAD. To explore which of these three lipoproteins are risk factors for causing CAD, we designed an in-depth Mendelian randomization study in the four aspects: (1) select two SNP sets respectly from two independent lipid-CAD GWAS datasets so that the MR results are supported by each other; (2) multivariate MR (MVMR) analysis is performed by using total, shared, and unique (unshared) SNP sets^39^ as instrumental variables in which pleiotropic effects of SNPs among multiple factors leading to confounding causality can be detected; (3) perform multiple MR methods on the same data to exclude inference bias due to difference in methods; (4) the MR results are confirmed by simulation without pleiotropic effects. We here propose a standardized approach (Supplementary Notes) based on the empirical selection of Do *et al*.^3^ to select two different sets of genetic variants from two independent GWAS meta-analysis datasets. Voight^40^ proposed a simulation method to simulate data for MR analysis, but this method is not suitable to simulate data of GWAS meta-analysis results; we therefore developed a new method to do simulation test.

## Results

### Selection of SNPs

**Figure 2:**
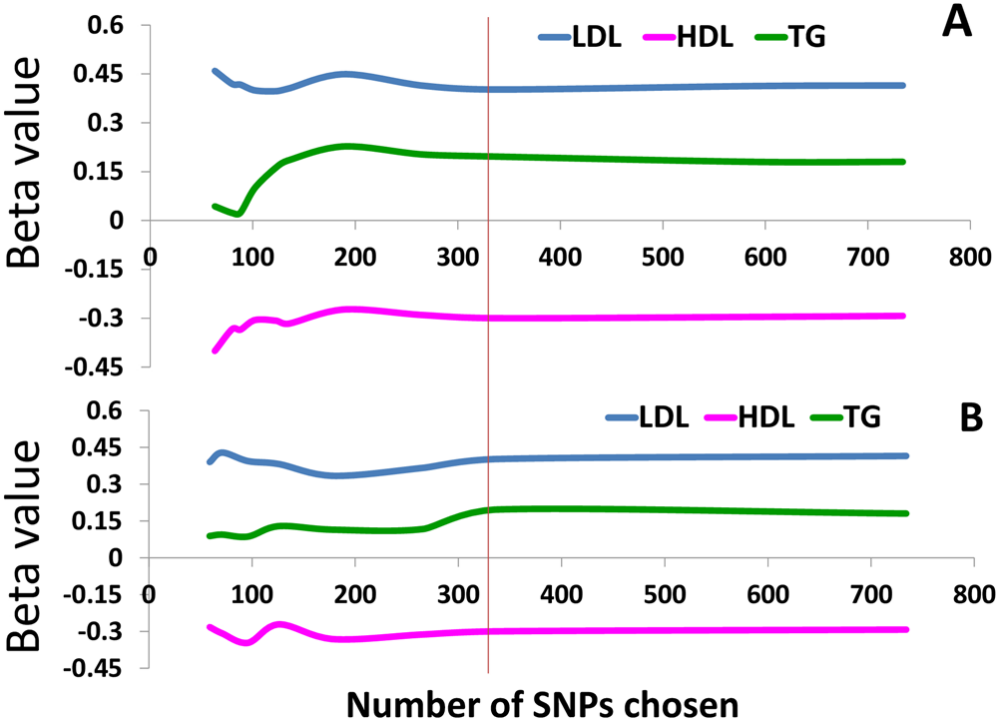
Relationship between *β*-values of CAD risk factors and number of selected SNPs. Linear regression coefficients (*β* values) of three lipid components on CAD were linearly plotted along numbers of SNPs chosen with *P*_*v*_, *P*_*c*_, *P*_*d*_ and adjacent interval length (AIL) between SNPs. Scheme A: SNPs were chosen with AIL =25, 20, 15, 10, 5, 1 kbp after setting *P*_*v*_ = 5*e*^−8^, *P*_*c*_ = *P*_*d*_ =0.99, …, *P*_*c*_ = 0.95, *P*_*d*_ = 0.972 (see Supplementary Table S2 and Methods). Scheme B: SNPs were chosen with AIL =25, 20, 15, 10, 5, 1 kbp after setting *P*_*v*_ = 5*e*^−8^, *P*_*c*_ = *P*_*d*_ = 0.979, …, *P*_*c*_ = 0.95, *P*_*d*_ = 0.972 (see Supplementary Table S2 and Methods).

**Figure 3:**
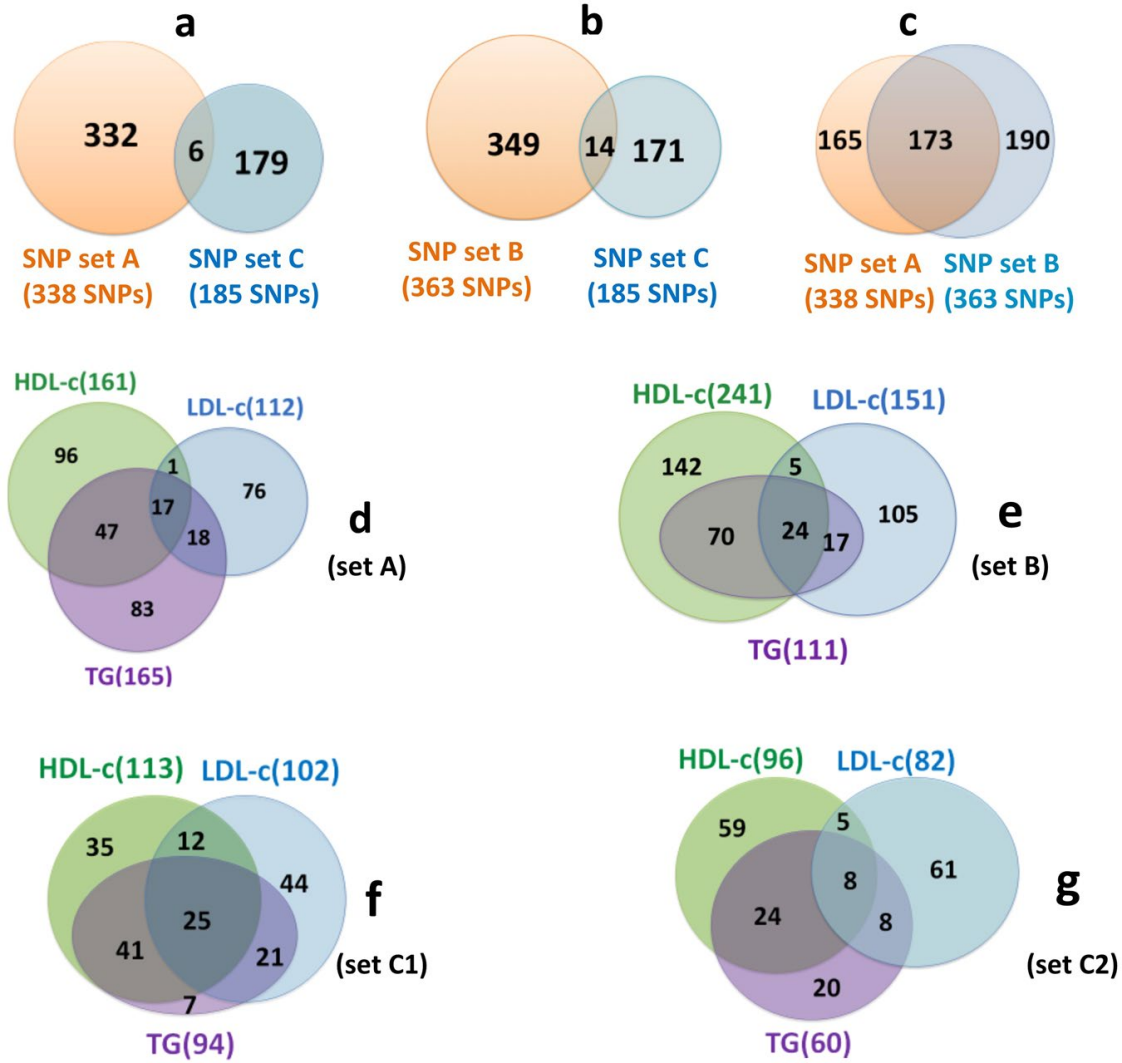
Venn diagrams of SNPs associated with lipid components. (**a**) Overlap between 185 SNPs selected by Do *et al*.^3^ (set C) and 338 SNPs (set A) selected by us from Mc-lipid-CAD data. (**b**) Overlap between set C and 363 SNPs (set B) selected by us from jointGwasMc-lipid-CAD data. (**c**) Overlap between set A and set B. (**d**) Overlap among LDL-c, HDL-c and TG associated SNPs (p = 5e-08) in set A. (**e**) Overlap among LDL-c, HDL-c and TG associated SNPs (p = 5e-08) in set B. (**f**) Overlap among LDL-c, HDL-c and TG associated SNPs (p = 1e-03) in set C, labelled as C1. (**g**) Overlap among LDL-c, HDL-c and TG associated SNPs (p = 5e-08) in set C, labelled as C2.

We selected multiple sets of SNPs (Supplementary Table S2) by changing the selection thresholds and by performing our selection method on the Mc-lipid-CAD data that contain 78,112 SNPs (Supplementary Table S1 and Methods). We then performed multivariate linear regression of lipid components on these datasets. The results show that *β* estimates of the three lipid components fluctuate with changes in the number of SNPs selected (Supplementary Table S2) but all converged at a data point with 338 SNPs (Fig. 2A,B), suggesting that SNPs selected with adjacent interval length, AIL > 1 kbp and/or *P*_*d*_ > 0.979 and *P*_*c*_ > 0.979 could give unstable and uncertain estimates of causal effects, and the 338 SNPs selected with *P*_*d*_ = *P*_*c*_ = 0.979 may provide enough and unbiased instrumental information for causal inference on CAD. By using a similar way and *P*_*v*_ = 5e-08, *P*_*c*_ = 0.984 and *P*_*d*_ = 0.985, we selected a second set of 363 SNPs from another dataset, jointGwasMc-lipid-CAD, that contain 2,436,375 SNPs (Supplementary Table S1 and Methods). For consistency, hereinafter, we refer to datasets of 338 and 363 SNPs selected respectively from Mc-lipid-CAD and jointGwasMc-lipid-CAD as datasets A and B (Supplementary data: dataset A and dataset B). Datasets A and B have 173 common SNPs (Fig. 3c), while they only have 6 and 14 common SNPs, respectively, with the SNP set provided by Do *et al*.^3^ (similarly, we refer to the dataset of the 185 SNPs as dataset C) (Fig. 3a,b). MR-PRESSO^41^ analysis did not find any outliers in dataset A at p-value <0.35 (Supplementary Table S4), but three SNPS from dataset B were found to be outliers. We created SNP data B’ by removing those three SNPs from dataset B (Supplementary Tables S9). Because MR analysis shows that SNP datasets B and B’ do not have essential differences in the evaluated associations of three lipids with CAD (Supplementary Table S9), datasets A and B are valid for inferring the causality of lipid on CAD. In addition, we used definition of White *et al*.^15^ to calculate R^2^ values of the three lipid components in six different SNP datasets (Supplementary Table S6) and found that R^2^ of the three lipid components had larger difference among them and also between datasets A and B for shared and unique SNPs, suggesting that the variance is due to genetic factor and was not correlated with inference of risk factors for CAD.

### Associations of SNPs selected with multiple potential risk factors for CAD

Among the 338 SNPs, only 17 SNPs were commonly associated with all the three lipid components, while 96, 76, and 83 SNPs were uniquely associated with HDL-c, LDL-c, and TG, respectively, at *p* ≤ 5e-08 (Fig. 3d). Similarly, from dataset B, only 14 SNPs were common to all these three lipid components, while 142 and 105 SNPs were uniquely associated with HDL-c, LDL-c, respectively. No SNP was found to be associated with TG at *p* ≤ 5e-08 (Fig. 3e). Interestingly, both HDL-c and LDL-c have very few common SNPs (Fig. 3d–f). This observation is in congruence with the common SNPs found in the SNP dataset C of Do *et al*.^3^ at p ≤ 5e-08 (Fig. 3g, recreated using data from Supplementary Fig. 3 of Do *et al*.^3^), suggesting that these two lipid components are independent of each other. In datasets A and B, TG shared 64 and 94 SNPs with HDL-c, 35 and 41 SNPs with LDL-c, respectively (Fig. 3d,e). These demonstrate that Mc and jointGwasMc lipid data are two very different lipid data.

### Single-variable MR inference of lipid risk for CAD

**Figure 4:**
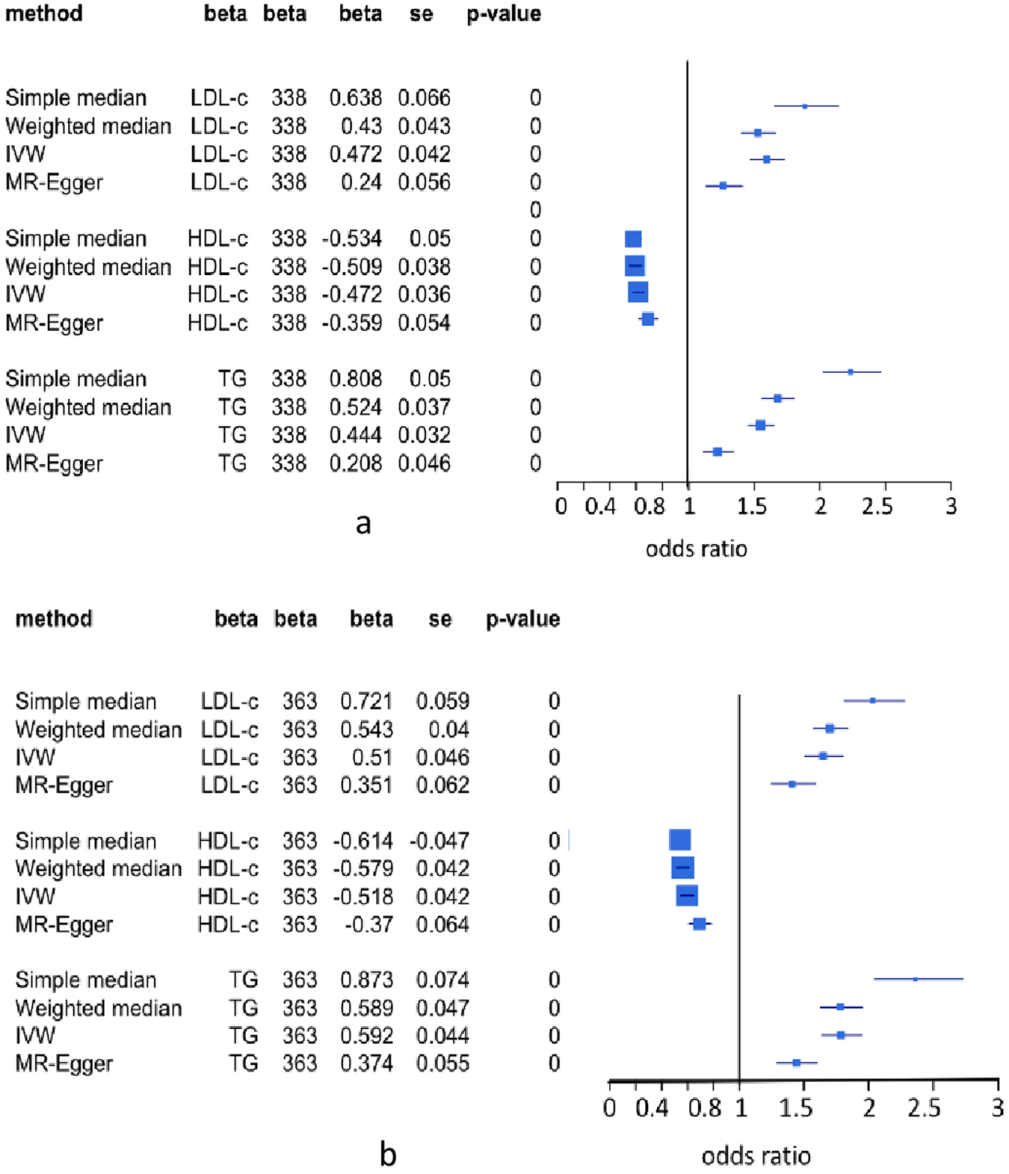
Results of different single-variable MR methods for testing associations of lipid components with risk for CAD. IVW is inverse variance weighted. MR-Egger is single-variable regression on outcome via adjusting intercept for pleiotropy of SNPs associated with exposure and outcome. Simple median-based method is simple linear regression of single-variable on outcome to estimate *β*-value and statistics for association of causal variable with risk for outcome. Weighted median-based method also is linear regression approach to estimate *β* and statistics for association causal variable with risk for outcome but uses weight as penalization to calculate the median of the ratio instrumental variable. (**a**) 338 SNPs were selected from Mc-lipid-CAD data. (**b**) 363 SNPs were selected from jointGwasMc-lipid-CAD data.

**Figure 5:**
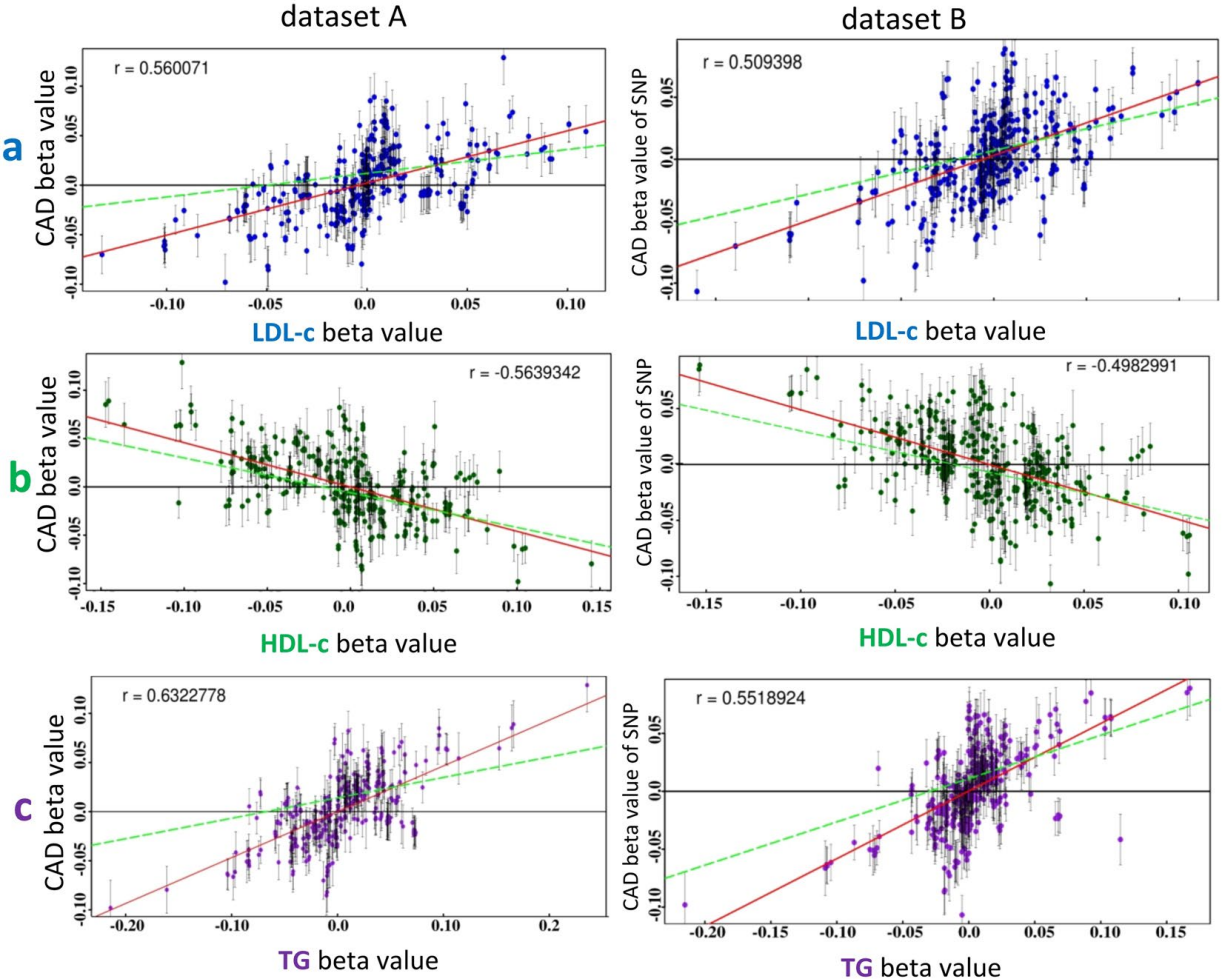
Scatter error-bar plots of lipoprotein-cholesterols versus CAD. Panels a, b, and c are respectively scatter error-bar plots of associations of SNPs with LDL-c, HDL-c, and TG versus those with risk for CAD based on dataset A from Mc-lipid-CAD data and dataset B from jointGwasMc-lipid-CAD. Red solid line is a general regression line and green dash line is an MR-Egger regression line.

To counter the inference bias of a single-variable MR method, we performed four single-variable MR methods on datasets A and B. These methods include simple median-based, weighted median-based, inverse-variance-weighted (IVW) and MR-Egger^20,38^. Results indeed show significant difference in the *β* estimates of lipids among these methods but they consistently inferred that LDL-c and TG were associated with increasing risk for CAD, while HDL-c was associated with reducing risk for CAD at p = 0.0 (Fig. 4a,b), which are consistent with clinical observations that HDL-c has a strong protection effect of against CAD^8,9,11,13,16^. These results can also be well explained by the scatter error-bar plots of associations of SNPs with LDL-c (Fig. 5a), HDL-c (Fig. 5b), and TG (Fig. 5c) versus *β* values of these SNPs associated with CAD in datasets A and B. LDL-c and TG were positively correlated with CAD risk (*r* > 0.5, *p* <0.0001) (Fig. 5a,c), while HDL-c was negatively correlated with CAD risk (*r* <−0.49, *p* <0.0001) (Fig. 5b).

### Multivariate MR (MVMR) inference of lipid risk for CAD

**Figure 6:**
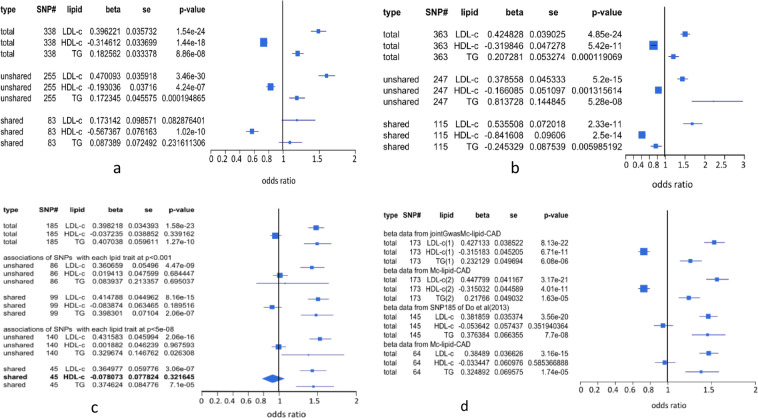
MVMR inference of associations of lipid components with risk for CAD. Unique SNPs, also called restricted SNPs^39^, consist of SNPs associated with only one lipid component. Shared SNPs are SNPs commonly associated with two or all of three lipid components. P-value is p-value for genetic association of a lipid component with CAD. Std is standard deviation. 95% confidence interval of  odds ratio of each lipid component on CAD was given in the forest plot. (**a**) 338 SNPs selected from Mc-lipid-CAD data were dissected into a set of 83 shared SNPs and a set of 255 unique SNPs. (**b**) 363 SNPs selected from jointGwasMc-lipid-CAD data were dissected into a set of 116 shared SNPs and a set of 247 unique SNPs. (**c**) 185 SNPs selected by Do *et al*.^3^ were dissected into a set of 99 shared SNPs and a set of 86 unique SNPs defined under p-value =0.001(see Do *et al*.^3^), a set of 45 shared SNPs, and a set of 140 unique SNPs defined under p-value = 5e-08. (**d**) 173 SNPs are common SNPs between 338 SNPs selected from Mc-lipid-CAD data and 363 SNPs selected from jointGwasMc-lipid-CAD. For 173 SNPs, data for LDL-c(1), HDL-c(1) and TG(1) come from dataset B and data for LDL-c(2), HDL-c(2) and TG(2) come from dataset A. 145 SNPs were re-selected by using White *et al*.'s method^15^ from 185 SNPs of Do *et al*.^3^. 64 SNPs were common SNPs between the 185 SNPs of Do *et al*.^3^ and 78112 SNPs in Mc-lipid-CAD data and the data for LDL-c, HDL-c and TG come from data of 185 SNPs.

Since single-variable MR cannot address statistical bias due to covariation among multiple risk variables, we performed MVMR on dataset A to adjust for co-variables and found that both LDL-c and TG were positively associated with risk for CAD (*p* =1.54e-24, and *p* = 8.86e-08), HDL-c was negatively associated with risk for CAD (*p* = 1.44e-18) (Fig. 6a). To corroborate this result, we also performed MVMR on dataset B (Fig. 6b), datasets D1 and D2 (dataset of 173 SNPs derived from dataset A is referred to as dataset D1 and from dataset B as dataset D2, see Supplementary data) (Fig. 6d) and observed very similar results. Compared to the results shown in Fig. 4, MVMR analysis made more precise statistical inferences, yet both analyses reached the same conclusions: high levels of LDL-c and TG in blood increase the risk for CAD, while high level of HDL-c decreases the risk for CAD. However, MVMR analyses of data C^3^, dataset E^15^ (145 SNPs selected from dataset of 185 SNPs, see Methods), and dataset F (46 common SNPs between dataset C and Mc-lipid-CAD, see Methods) also had similar results: LDL-c and TG were associated with increasing risk for CAD but HDL-c was not a risk factor causing CAD (Fig. 6c,d). It is, however, not surprising that this is due to the fact that the datasets E and F are subsets of dataset C.

### MR inference of lipid risk for CAD using shared and unique SNPs

**Figure 7:**
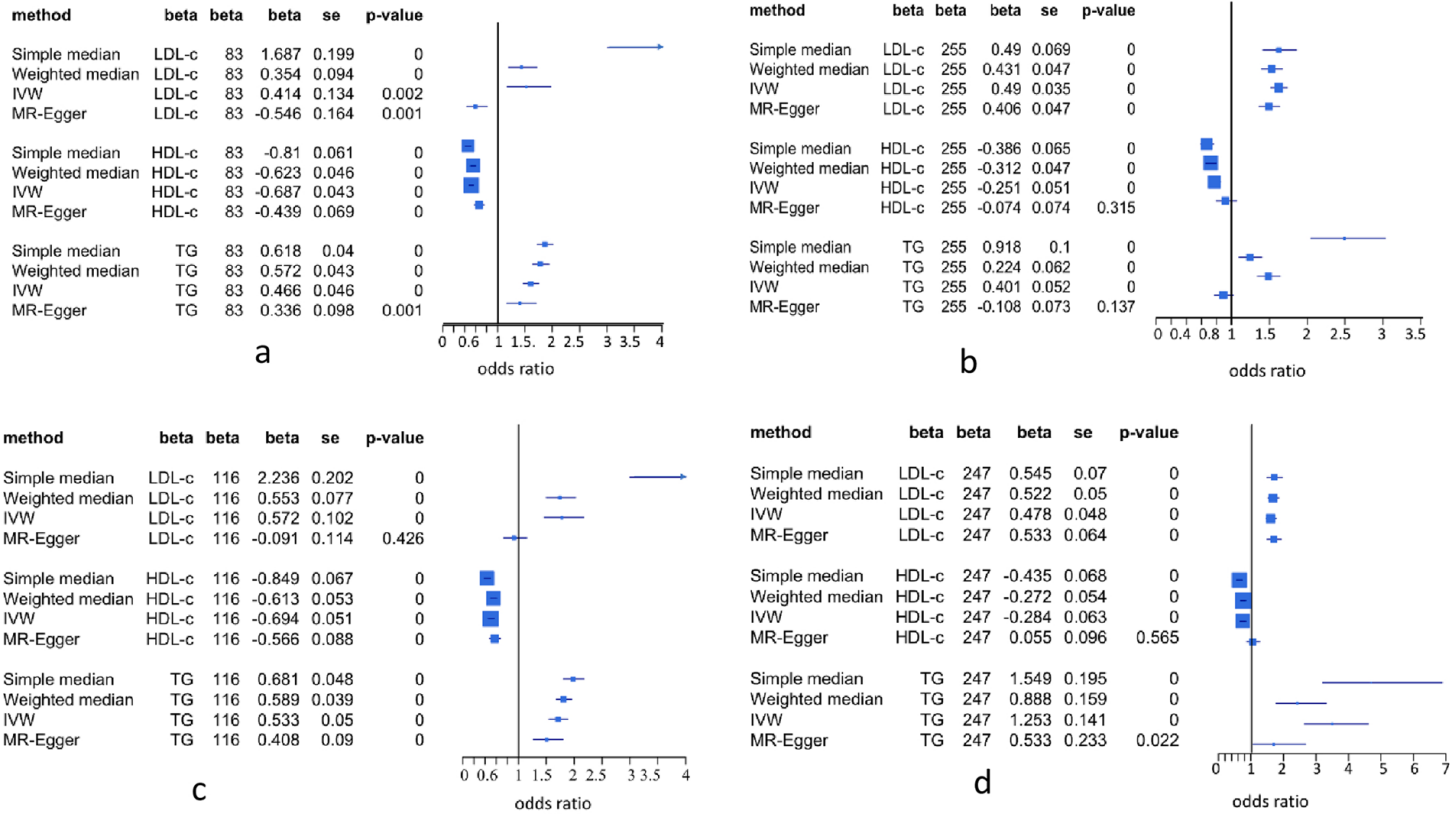
Associations of LDL-c, HDL-c and TG with CAD estimated by using different single-variable MR methods. IVW is simple regression method but uses inverse-variance weighted to estimate *β* (causal effect) on outcome^20^. MR-Egger is Egger regression of causal variable on outcome via adjusting intercept for pleiotropy of SNPs associated with exposure and outcome^20^. Simple median-based method is simple linear regression of single-variable on outcome to estimate *β* value and statistics for association of causal variable with risk for outcome. Weighted median-based method also is linear regression approach to estimate *β* and statistics for association causal variable with risk for outcome but uses weight to penalize median. *β* is coefficient of regression of lipid on a disease (CAD), a causal (risk) effect of causal variable on the disease (CAD). Std is standard deviation of *β* and p-value is for t-testing association of causal variable with risk for disease. 95% confidence interval of odds ratio  of each lipid component on CAD was given in the forest plot. (**a**) 83 shared SNPs in 338 SNPs; (**b**) unique 255 SNPs in 338 SNPs; (**c**) 116 shared SNPs in 363 SNPs, (**d**) 247 unique in 363 SNPs.

Despite that multivariate MR can correct the estimation bias by adjusting for co-variables, it is not clear whether this bias is coming from pleiotropic effects of the shared SNPs or from the SNP selection bias or both. To distinguish SNP pleiotropy from SNP selection bias, we followed the analysis method of Holmes *et al*.^39^ by splitting SNP sets into shared and unique (unshared) SNP subsets (see Methods). We applied these four single-variable MR methods to the shared and unique SNP datasets, and found that except for MR-Egger, the other three methods all detected associations of LDL-c and TG with increasing risk for CAD, and protection effect of HDL-c against risk for CAD at p = 0.0 by using either shared SNPs (Fig. 7a,c) or unique SNPs (Fig. 7b,d) as instrumental variables. These results are highly concordant with the error-bar plots of association distributions of these shared SNPs (Supplementary Fig. S1) and unique SNPs (Supplementary Fig. S2) in LDL-c, HDL-c and TG versus those in CAD. MR-Egger analysis showed pretty inconsistent results (Fig. 7a for LDL-c, 7b for HDL-c and TG, 7c for LDL-c and 7d for HDL-c and TG). Supplementary Fig. S2a shows that MR-Egger regression lines (green dash lines) were greatly deviated against real distribution of associations of shared SNPs with LDL-c versus those with CAD in datasets A and B. We noted that intersection in all regressions of lipid components on CAD in datasets A and B was not significant (data not shown).

We separately performed MVMR on the shared and unique SNP datasets. The results demonstrated that using the 255 unique SNPs in dataset A as instrumental variables, LDL-c became more strongly associated (0.47, p = 3.46e-30) with increasing risk for CAD (Fig. 6a), but TG did not significantly change association with increasing risk for CAD, while increased *β*, HDL-c was still associated with reducing risk for CAD (p = 4.24e-07, Fig. 6a). Using the 247 unique SNPs in dataset B as instrumental variables, LDL-c was associated with increasing risk for CAD (p = 5.25e-15, Fig. 6b) and HDL-c was associated with reducing risk (p = 0.0013, Fig. 6b). However, TG had big *β* (=0.814) with *p* = 5.28e-08(Fig. 6b). As seen in Fig. 3e, no unique SNP was associated with TG under *p* = 5e-08 and Supplementary Fig. S1c showed that TG had significantly correlation (r = 0.526, p < 0.0001) with CAD but compared to Fig. 5c, *β* values of SNPs in regression on TG in the unique SNP dataset were very close to zero. Hence, this strong association of TG with increasing risk for CAD was completely false under definition of p = 5e-08. As shown in Fig. 6c, performing MVMR analysis on the 86 unique SNPs defined at *p* < 0.0001 in dataset C, LDL-c was significantly associated with growing risk for CAD, but TG and HDL-c were not associated with the risk for CAD (Fig. 6c). The 83 shared SNPs from dataset A and 99 shared SNPs from dataset C had opposite MVMR results (Fig. 6a,c), indicating that in dataset C the association of TG with increasing risk for CAD was derived from instrument of the 99 shared SNPs with pleiotropic effects. To verify this point, we redefined associations of SNPs with LDL-c, HDL-c and TG under *p* = 5e-08 and got a new association distribution of these SNPs among three lipid components (Fig. 3g). Fifty four shared SNPs in Fig. 3f were moved to the unique SNPs, thus 140 of the 185 SNPs became unique. The MVMR analysis shows that TG was positively associated with risk for CAD at significant level of 0.05 (Fig. 6c). Using the 45 shared SNPs as instrumental variables, the results observed with the 99 shared SNPs were also obtained (Fig. 6c).

In dataset B, 116 shared SNPs as instrumental variables showed very strong association of LDL-c (*p* = 2.33e-11, Fig. 6b) with increasing risk for CAD but HDL-c and TG were strongly associated with reducing risk for CAD (Fig. 6b). As seen in Fig. 3d,e, HDL-c shared very small part of SNPs (18 SNPs in dataset A and 29 in dataset B) with LDL-c. Supplementary Table S3 also shows that HDL-c was not correlated with LDL-c in datasets A and B. Therefore, the strong association of HDL-c with reducing risk for CAD cannot be attributed to pleiotropic effects of those SNPs associated with LDL-c. Although TG had a lot of common SNPs (64 in dataset A and 94 in dataset B) with HDL-c and had very significant negative correlation with TG (Supplementary Table S3) so that association distribution of these shared SNPs in CAD was very significantly and positively correlated with their distribution in TG (*r* > 0.72, Fig. S3c), TG was not associated with CAD risk in dataset A or was associated with reducing risk for CAD in dataset B (Fig. 6a,b). The only explanation of these results is that these shared SNPs are instrumental variables for HDL-c only and TG is a confounding factor for CAD (see Discussion).

**Figure 8:**
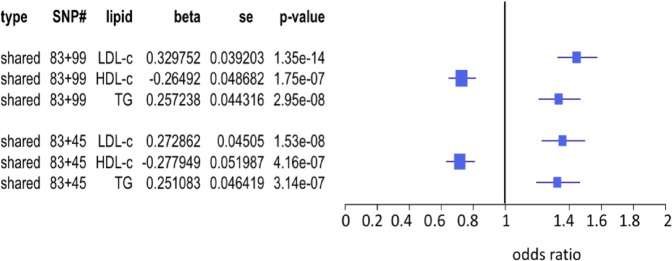
MVMR analysis of lipid risk for CAD using two shared SNP sets. MVMR inference of associations of lipids with risk for CAD using two shared SNP sets as instrumental variables: 83 shared SNPs come from the dataset A, 99 and 45 shared SNPs from dataset C were respectively defined at p < 0.0001 and p < 5e-08.

We used 83 shared SNPs from dataset A (Fig. 6a) and 99 shared SNPs defined by Do *et al*.^3^ under p = 0.0001 from dataset C (Fig. 6c) as complementary instrumental variables for MVMR analysis. The result, as expected, shows that LDL-c and TG were associated with increasing risk for CAD (*p* = 0.331.35e-14 for LDL-c and *p* = 0.2572.95e-08 for TG) and HDL-c had protection against risk for CAD (*β* = −0.265, *p* = 1.75e-07) (Fig. 8). The similar result was obtained by using the 83 shared SNPs from dataset A and 45 shared SNPs defined at *p* < 5e-08 in the dataset C (Fig. 8). Therefore, no association of HDL-c with CAD in dataset C could be due to losing many SNPs as instrumental variables for association of HDL-c with CAD by selection bias.

### Simulation inference of lipid risk for CAD

Multiple SNPs selected as instrumental variables are inevitably subjected to the pleiotropy issue. Different from horizontal pleiotropic effect defined as co-association of a SNP with a trait and an outcome in MR-PRESSO^41^, here the pleiotropic effect refers to co-association of a SNP with two or more biological risk factors. This type of pleiotropic effect arises if a gene within which the SNPs are located has multiple functions or works for multiple traits or in the upstream of a pathway. To exclude the pleiotropic effects in MVMR inference, we conducted simulations to test associations of lipids with risk for CAD. Our simulation method was given in Methods. We used Mc-lipid-CAD dataset of 78112 SNPs as mother data and used normal distributions with means and variances of *β*_*LDL*_, *β*_*HDL*_, *β*_*TG*_, *β*_*CAD*_ over all these 78112 SNPs to randomly simulate *b*_*LDL*_, *b*_*HDL*_, *b*_*TG*_, *b*_*CAD*_ profiles of 78112 SNPs where *b* is null *β*. We separately created null allele profiles of SNPs for LDL-c, HDL-c, TG, and CAD by permuting the major alleles among these 78112 SNPs and used uniform distribution^42^ to create two null p-value profiles: 1e-07 ~ 1 for no noise to choose SNPs in a test data and 1e-12 ~ 1 for making noise to choose SNPs in a test data. We also simulated null sample sizes for each SNP in each lipid component and CAD using normal distributions with sample size mean ${\bar{N}}_{x}$ and variance ${\sigma }_{x}^{2}(N)$ over all SNPs where *x* = LDL-c, HDL-c, TG, or CAD. We then randomly assigned a set (*β*_*jx*_, *A*1_*jx*_ *N*_*jx*_ *β*_*jd*_, *A*1_*jd*_, *N*_*jd*_) of *SNP*_*j*_ (j = 1, …, m) in a test dataset of m SNPs to the null data and replace a set (*b*_*ix*_, *a*1_*ix*_ *n*_*ix*_, *b*_*id*_, *a*1_*id*_, *n*_*id*_) of *SNP*_*i*_ *(I* = 1, …, M) where *x* =LDL, HDL, or TG, and d = CAD; *β*, *A*1, and *N* are respectively real regression coefficient (*β*), major allele, and sample size for *SNP*_*j*_ in a test dataset and *b*, *a*1, and *n* are respectively null *β*, major allele, and sample size for *SNP*_*i*_ in the null dataset. This process was repeated until the last SNP in a test data. If m/M < 0.005, the probability that a test SNP is co-associated with two lipid components is 1.9e-05, therefore, none of the test SNPs in datasets A, B and C would be co-associated with two or all of the three lipid components. These test SNPs are independently and uniformly scattered on 22 chromosomes, and hence no pleiotropic effect of these test SNPs among lipids and CAD. However, relationships of a test SNP with a lipid component and with CAD in a test dataset are not changed in the simulated data. If a test SNP is associated with two or three lipid components, then this SNP is duplicated or triplicated to generate two or three SNPs, that is to say, these duplicated/triplicated SNPs are randomly scattered to the whole genome, thus each of them is associated with only one lipid component. We used our methods above to select SNPs from the simulation data. To test the association of lipids with CAD risk without the pleiotropic impacts in datasets A, B and C, we here used them as test datasets and set *P*_*v*_ = 5e-8 and *P*_*c*_ = *P*_*d*_ = {0.0, 0.7, 0.9, 0.915, 0.93} as SNP selection criteria for dataset C, *P*_*v*_ = 5e-08 and *P*_*c*_ = {0.9, 0.95, 0.97, 0.98, 0.99} and *P*_*d*_ = {0.97, 0.98, 0.99} for dataset A. We still conducted MVMR on the data selected from the simulated data. For each combination of *P*_*c*_ and *P*_*d*_, we repeated 10 simulations. We used averaged *β* and *p*-value (${\bar{\beta }}_{kx}\,{\rm{and}}\,{\bar{p}}_{kx}$) to test association of factor *x* with risk for CAD where k is the kth combination of *P*_*c*_ and *P*_*d*_, *x* = LDL, HDL, or TG. Our simulation results were summarized in Supplementary Tables S7–S9. For the first null *p*-value profile (*p*-value is distributed from 1e-07 to 1) and test dataset A, we found that given *p* < *P*_*v*_ = 5e-08, SNPs selected by using our method with all 15 *P*_*c*_–*P*_*d*_ combinations from simulated datasets were truly derived from the test dataset and all had FDR (false discovery rate) = 0 (Supplementary Table S7). Using these SNPs without pleiotropic effects as instrumental variables, MVMR analysis showed ${\bar{\beta }}_{LDL}$ = 0.482 ~ 0.426 with ${\bar{p}}_{LDL}$ = 3.83E-14 ~ 0.0144, ${\bar{\beta }}_{HDL}$ = −0.369 ~ −0.401 with ${\bar{p}}_{HDL}$= 2.4e-16 ~0.000155; ${\bar{\beta }}_{TG}$ = −0.039 ~ 0.023 with ${\bar{p}}_{TG}$ = 0.162 ~ 0.587 (Supplementary Table S7), suggesting that LDL-c was associated with increasing risk for CAD, HDL-c had a strong protection effect against CAD, and TG was not a risk factor causing CAD. Using dataset C as a test dataset, we found that given *p* < *P*_*v*_ = 5e-08, likewise, all SNPs selected by using our method with all 25 *P*_*c*_-*P*_*d*_ combinations from simulated datasets were truly derived from the test dataset and all had FDR = 0 for all *P*_*c*_-*P*_*d*_ combinations. MVMR analysis showed ${\bar{\beta }}_{LDL}$ = 0.365 ~ 0.402 with ${\bar{p}}_{LDL}$ = 2.98e-15 ~ 0.0085, ${\bar{\beta }}_{HDL}$ = −0.135 ~ −0.09 with ${\bar{p}}_{HDL}$ = 0.01489 ~ 0.20669 where 8 *P*_*c*_-*P*_*d*_ combinations had ${\bar{p}}_{HDL}$ < 0.05 and 17 had ${\bar{p}}_{HDL}$> 0.05 with number of SNPs associated with HDL-c < 75, ${\bar{\beta }}_{TG}$ = −0.332 ~ −0.264 with ${\bar{p}}_{TG}$ = 0.00015 ~ 0.1955 where 14 *P*_*c*_-*P*_*d*_ combinations had ${\bar{p}}_{TG}$ < 0.05 and 11 had ${\bar{p}}_{TG}$ > 0.05 with number of SNPs associated with TG < 36 (Supplementary Table S8), suggesting that LDL-c was associated with increasing risk for CAD but both HDL-c and TG were associated with decreasing risk for CAD when more than 75 SNPs associated with HDL-c and more than 36 SNPs associated with TG were selected. For the second null p-value profile (p-value is distributed from 1e-12 to 1), we set *P*_*c*_ = {0.9, 0.95, 0.97, 0.98, 0.99} and *P*_*d*_ = {0.9, 0.95, 0.97, 0.98} and used dataset A as a test dataset. We found that under *P*_*v*_ = 5e-08, SNPs selected by using 20 *P*_*c*_-*P*_*d*_ combinations had FDR = 0.06 ~ 0.136 and ${\bar{\beta }}_{LDL}$ = 0.215 ~ 0.237 with ${\bar{p}}_{LDL}$ = 0.00402 ~ 0.0351, ${\bar{\beta }}_{HDL}$= −0.226 ~ −0.191 with ${\bar{p}}_{HDL}$ = 0.00078 ~ 0.04489, ${\bar{\beta }}_{TG}$ = −0.0606 ~ −0.0232 with ${\bar{p}}_{TG}$ = 0.3864 ~ 0.6061 (Supplementary Table S7), suggesting that null SNPs selected impacted *β* estimation and *p*-values, and the conclusion was the same with that in the case of null p-value profile from 1e-07 to 1. By using dataset C as a test dataset, SNPs selected by all *P*_*c*_-*P*_*d*_ combinations {(0.0, 0.0), …, (0.9,0.9)} had FDR = 0.31 ~ 0.327, which indicates that number of the false SNPs selected is not related to *P*_*c*_ and *P*_*d*_ values. This is because dataset C does not contain sample size for each SNP in each lipid component and in CAD. Compared to the results obtained by using dataset A as the test dataset (Supplementary Table S7), we found that if GWAS lipid-disease data have samples size data, valid SNPs would be selected by using *P*_*c*_ and *P*_*d*_ under *P*_*v*_ = 5e-8. In addition, compared to the results obtained from the case of null p-value profile of 1e-07 to 1, we also found that even though FDRs were large, MVMR results were not significantly changed (Supplementary Table S8). This may be because the true SNPs associated with LDL-c and/or TG had larger *β*-values and stronger relationship with CAD than the false SNPs selected.

To verify the results obtained from dataset A, we used dataset B as a test dataset with *P*_*v*_ = 5e-08, *P*_*c*_ = {0.0, 0.05, 0.7}, and *P*_*d*_ = {0.0, 0.7, 0.9, 0.93} as SNP selection criteria. The test results show that SNPs selected by using *P*_*c*_-*P*_*d*_ combinations (0.0, 0.0) ~ (0.5, 0.93) had different FDR values, but numbers of SNPs associated with LDL-c, HDL-c and TG were not changed. All SNPs selected by using *P*_*c*_-*P*_*d*_ combinations (0.7, 0.0) ~ (0.7,0.93) were truly derived from the test dataset and hence had FDR = 0 (Supplementary Table S9). Compared *β*-values and p-values between these two *P*_*c*_-*P*_*d*_ combination groups, we found that associations of lipid components with risk for CAD were not significantly changed by false SNPs. Supplementary Table S9 shows that ${\bar{\beta }}_{LDL}$= 0.2839 ~ 0.2705 with ${\bar{p}}_{LDL}$ = 8.46e-07 ~ 2.85e-06, ${\bar{\beta }}_{HDL}$ = −0.2316 ~ −0.2144 with ${\bar{p}}_{HDL}$ = 0.00181 ~ 0.00276, and ${\bar{\beta }}_{TG}$ = − 0.075 ~ −0.0661 with ${\bar{p}}_{TG}$ = 0.2446 ~ 0.2731. These results were similar to those obtained by testing dataset A in the cases of null p-values from 1e-12 to 1.

## Discussion

Using shared, unique and total SNPs as instrumental variables, LDL-c was mostly found to be associated with increasing risk for CAD in datasets A and B by either single-variable MR methods or MVMR. This observation is well consistent with all clinical observations. Similarly, HDL-c was always found to be associated with reducing risk for CAD in these two SNP datasets. This finding is also well consistent with clinical observations^8,9,11,13,16^ but conflicted with the findings of Do *et al*.^3^, Bowden *et al*.^20,38^ and White *et al*.^15^. Re-analysis of their SNP datasets showed that HDL-c is not a CAD risk factor because some SNPs that could be used as instrumental variables for association of HDL-c with CAD (Fig. 8) were lost. Association of TG with risk for CAD is not consistent in datasets A and B when shared SNPs were used as instrumental variables (Fig. 6a,b). In addition, using the unique SNPs as instrumental variables, TG had *β*_*TG*_ = 0.817 with *p*_*TG*_ = 5.28e-08 in dataset B. Supplementary Fig. S1c shows that *β*_*TGj*_ was very small (−0.02 to 0.02), indicating that strong association of TG with increasing risk for CAD is completely due to the association of SNPs with LDL-c and TG that were not selected under *p* = 5e-08 were stronger than those with HDL-c and TG. Association of TG with increasing or decreasing risk for CAD was dependent on balance status between associations of SNPs selected with LDL-c and TG and those with HDL-c and TG (see Supplementary Note S3 for detail).

Linkage disequilibrium (LD) effect has been believed to be excluded in MVMR analysis. For example, dataset C of Do *et al*.^3^ was selected without LD effect (D’=0 for each SNP pair). However, there is a big SNP selection bias in a LD block interval in which many SNPs are located with D’ > 0 because anyone of these SNPs selected within a LD block interval (or a gene) has D’ = 0 with one of SNPs in another LD block interval. As we know, all selected SNPs are associated with at least one potential risk factor under p-value = 5e-08 but not necessarily associated with the disease of study; hence, association of a potential risk factor with the disease is not related with LD and selection of SNPs without LD may lead to loss of SNPs that are required for instrumental association of a potential risk factor with a disease of study and are irreproducible. A typical example is that in dataset C, 185 SNPs selected with D’=0 has a big selection bias and cannot be used as instrumental variables for detecting association of HDL-c with CAD risk because many SNPs that associate HDL-c with CAD risk were missed out.

Like MR-Egger^20^, MR-PRESSO^41^ also addresses the horizontal pleiotropic issue that one SNP is associated not only with a potential risk factor but also with a disease of study in MR analysis. Although MR-PRESSO is an MVMR method, MVMR itself cannot estimate pleiotropic effects of SNPs on causal inference of the disease of study. Therefore, MR-PRESSO also cannot be applied to address such a pleiotropic issue. Comparing total SNP sets to the shared SNP sets in Fig. 6, one can find that these types of pleiotropic effects might result in incorrect statistical inference of causality of the disease of study. In our current study, we used the shared SNPs as instrumental variables to address pleiotropic effects on the disease causality; however, the shared SNPs are dependent on the p-value threshold. To avoid the threshold issue of shared SNPs, we are planning to develop a new statistical method by applying Write’s path analysis^43,44^ to dissect causal effect into direct and indirect effects. The direct causal effect comes from association of a potential risk factor itself with the disease of study while indirect causal effects are defined as pleiotropic effects.

It is necessary to discuss the results of Frikke-Schmidt *et al*.^25^, Voight *et al*.^14^ and Zanoni *et al*.^45^. Frikke-Schmidt *et al*.^25^ found that HDL-c-associated loss-of-function mutations in the ABCA1 gene do not increase the risk of ischemic heart disease. Voight *et al*.^14^ reported that LIPG 396Ser associated with HDL-c are not associated with risk for CAD, while Zanoni *et al*.^45^ found that variant P376L of SCARB1 associated with high HDL-c in blood had a significantly higher risk of coronary heart disease (CHD). The results of Frikke-Schmidt *et al*.^25^ and Voight *et al*.^14^ are expected in MR analysis. All of the 338 SNPs associated with HDL under p < 5E-08 are not associated with CAD (p > 5E-08) (see Supplementary datasets). However, HDL-c beta profile of the 338 SNPs was associated with CAD beta profile of these SNPs (Fig. 5b). The variant P376L of SCARB1 is just a horizontal pleiotropic variant associated with HDL-c and TG and also with CHD and hence cannot be used as instrumental variable for MR analysis.

MVMR analyses of datasets A and B demonstrate that our SNP selection method is suitable for accurate multiple MR analysis of GWAS-wide data obtained from large samples. However, its accuracy would become low as sample size decreases because Pc and Pd depend on the maximum sample size among SNPs. Hence, our method is not suitable for SNP selection if the sample size is small.

## Methods

### Data collection

Four datasets were used in our current study. They are (1) metabochip analysis data (Mc) of lipoprotein cholesterols, LDL-c, HDL-c, and TG; (2) joint GWAS and metabochip analysis data (jointGwasMc) of lipoprotein cholesterols, LDL-c, HDL-c, and TG. Data Mc and jointGwasMc were downloaded from Willer *et al*.^46^. Each of these three lipoprotein cholesterols has a table listing the results of the meta-analysis: SNPID, estimate of regression coefficient (beta), sample size (individual number), major and minor alleles, and p-value for association of a SNP with blood lipid trait in the 1000 Genomes European samples. The GWAS SNP sets have been imputed; (3) GWAS meta-analysis data of CAD containing SNPID, reference allele frequency, log odds ratio (logOR), sample size and p-value etc. columns; (4) dataset of 185 SNPs selected by Do *et al*.^3^. The basic information and references or sources of these datasets were summarized in Supplementary Table S1. Since all the datasets used in our study are available in the public domain, we have not sought specific ethical reviews and/or consents from participants from the original studies.

### SNP selection for MRMV analysis of lipoprotein cholesterol risk for CAD

To select a set of valid SNPs as instrumental variables for MVMR analysis, we designed schemes A and B (see Supplementary Table S1). In scheme A, we first merged GWAS lipid data (Mc) and CAD data (CARDIoGRAMplusC4D) using SNPID to create a new dataset called Mc-lipid-CAD containing 78,112 SNPs. *P*_*vj*_ is defined as the smallest p-value among the three lipid components (LDL-c, HDL-c, and TG) for the j^th^ SNP. First, we conducted SNP selection from Mc-lipid-CAD data using a threshold, *P*_*vj*_ ≤ *P*_*v*_ = 5e-08 (Supplementary Note S1). These selected SNPs were associated with at least one of three lipid components under *P*_*v*_ = 5e-08 but not necessarily associated with CAD. We then defined *P*_*cj*_ as proportion of the total sample size for SNP_j_ to the largest total sample size across all SNPs in all causal variables of study. *P*_*dj*_ was similarly defined in CAD (Supplementary Note S1). We used criteria *P*_*cj*_ > *P*_*c*_,and *P*_*dj*_ > *P*_*d*_ to perform the second selection in the first-selected SNP set where *P*_*c*_ and *P*_*d*_ were given thresholds (or cutoffs). To explore if there is a linkage disequilibrium significantly impacting on causal analysis among chosen SNPs, we performed the third selection from the second-selected SNP set with SNP adjacent interval length (AIL) ≥ {25 kbp, 20 kbp, 15 kbp, 10 kbp, 5 kbp, 1 kbp} using our algorithm (Supplementary Note S2). In SNP selection scheme A, we selected SNPs from the first-selected SNP set by setting *P*_*c*_ = 0.95 and *P*_*d*_ = 0.972, *P*_*c*_ = *P*_*d*_ = 0.972, *P*_*c*_ = *P*_*d*_ = 0.979, *P*_*c*_ = *P*_*d*_ = 0.98, *P*_*c*_ = *P*_*d*_ = 0.99, respectively (Supplementary Table S1). In SNP selection scheme B, we selected SNPs from the first-selected SNP set using *P*_*c*_ = *P*_*d*_ = 0.972 in scheme A, *P*_*c*_ = *P*_*d*_ = 0.979 and did the third selection in the second-selected SNPs using AIL ≥ {25 kbp, 20 kbp, 15 kbp, 10 kbp, 5 kbp, 1 kbp} (Supplementary Table S1).

### SNP selection from jointGwasMc and CARDIoGRAMplusC4D data

To confirm our selection of SNPs in Mc-lipid-CAD data, we merged another GWAS lipid dataset, jointGwasMc, and a CAD dataset, CARDIoGRAMplusC4D (Supplementary Table S1) using SNPID to form a new dataset, called jointGwasMc-lipid-CAD data, that contain 2,436,375 SNPs. So jointGwasMc-lipid-CAD data are different from Mc-lipid-CAD data. By following SNP selection steps as described above, we selected 363 SNPs from jointGwasMc-lipid-CAD data using P_*v*_ = 5e-08, *P*_*c*_ = 0.984 and *P*_*d*_ = 0.985 and retrieved dataset B from this lipid-CAD data by using 363 SNPID (Supplementary data).

### Selection of SNP datasets

From Mc-lipid-CAD data of 78,112 SNPs, we selected 338 SNPs according to beta curves converging to number point of selected SNPs given in Fig. 2 and selected 363 SNPs from jointGwasMc-lipid-CAD using similar method. Venn-diagram analysis showed that 173 SNPs are common for both datasets. To explore if difference in data sources results in MR inference bias of lipid risk for CAD, we retrieved two different datasets of 173 SNPs (beta values, beta SD, alleles, allele frequency, p-value, individual number, etc.), respectively, from datasets A and B by using their SNPIDs (see Supplementary data). We also selected 145 SNPs from Do *et al*.’s 185 SNPs^3^ by performing the method of White *et al*.^15^. In addition, 64 SNPs were common SNPs between the 185 SNPs of Do *et al*.^3^ and 78112 SNPs in Mc-lipid-CAD data and the data for LDL-c, HDL-c and TG come from data of 185 SNPs.

### Shared and unique SNP data subsets

To detect the effects of SNP pleiotropy, we created two sets of SNPs for each dataset based on the relationship for each SNP with one or more risk factors of study. SNPs that are associated with more than one risk factors are categorized as shared while those associated with only one risk factor are called unique or non-pleiotropic using the following procedure:

Step1: Select SNPs associated with LDL-c using *p*_*LDL*_ <= 5e-08 from a selected dataset.

Step2: Select SNPs associated with HDL-c using *p*_*HDL*_ ≤ 5e-08 from the selected dataset.

Step3: Select SNPs associated with TG using *p*_*TG*_ ≤ 5e-08 from the selected dataset.

Step 4: Perform Venn-diagram analysis on these three SNP subsets and to respectively get SNPs associated with LDL-c only, with HDL-c only, and with TG only, then put them together to form a subset called unique SNPs. The unique SNPs are also called restricted SNPs^39^. The remained SNPs that are associated with two or all of the three risk factors are called shared SNPs with potential pleiotropic effects.

Step 5: Retrieve data (beta values, beta SD, alleles, allele frequency, p-value, individual number, etc.) for shared and unique SNPs from the two datasets described above.

### Simulation method for selection of SNPs

To validate our selection of SNPs and to study SNP pleiotropic effects on MR inference, we carried out a simulation study. Here we developed a new simulation method to test causal effects of lipids on CAD using GWAS lipid meta-analysis and GWAS CAD meta-analysis datasets. We used a lipid-CAD dataset as mother data where each lipid component has beta, p-value, individual number, major and minor allele columns and GWAS disease data also have beta (log odds), p-value, individual numbers in the case and control, major (reference) allele and minor (other) allele columns. We used uniform distribution^43^ to create null p-value profiles for null SNP associations with each lipid component and CAD. In each lipid component or in CAD, we respectively permutated major and minus alleles among all SNPs and used normal distributions with beta mean $(\overline{\beta })$ and variance over all SNPs to simulate a beta set for each lipid component and CAD. We then used a dataset of SNPs selected as a test dataset to test for association of a lipid component with risk for CAD. Relationships of SNPs with lipid components and CAD are given in the test dataset. We simulated null sample sizes for each SNP in each lipid component and CAD using normal distributions with sample size means ${\bar{N}}_{x}$ and variance ${\sigma }_{x}^{2}(N)$ over all SNPs where *x* = LDL-c, HDL-c, TG, or CAD. Then, we randomly assigned set (*β*_*jx*_, *A*1_*jx*_ *N*_*jx*_ *β*_*jd*_, *A*1_*jd*_, *N*_*jd*_) of *SNP*_*j*_ (j = 1, …, m) from a test dataset of *m* SNPs to the null data and replaced a set (*b*_*ix*_, *a*1_*ix*_ *n*_*ix*_, *b*_*id*_, *a*1_*id*_, *n*_*id*_) of *SNP*_*i*_ *(i* = 1, …, M) in the null data where *x* = LDL, HDL, or TG and d = CAD, *β*, *A*1, and *N* are real beta, major allele, and sample size for *SNP*_*j*_ in a test dataset and *b*, *a*1, and *n* are null *β*, major allele and sample size for *SNP*_*i*_ in the simulated null dataset and repeated until the last SNP in the test dataset. Here each *x* was independently assigned to the null data. The test SNPs were also independently and uniformly assigned to 22 chromosomes, hence there was no pleiotropic effect of SNPs among lipid components. However, relationships of a test SNP with a lipid component and CAD in a test dataset were not changed in the simulated data. We use our methods above to select SNPs from the simulation data. To fully test for associations of lipids with risk for CAD without impacts of pleiotropic and LD effects in a test dataset, we need to set *P*_*v*_ =5e-08, *P*_*c*_ vector and *P*_*d*_ vector as criteria to select SNPs. Our method conducts MVMR analysis on the data selected from the simulated data. To reduce fluctuation of beta for testing association of a lipid component with risk for CAD, our method can conduct replication simulations for SNP selection by using each combination of *P*_*c*_ and *P*_*d*_. Our method would output averaged total SNP number, false SNP number, numbers of SNPs associated with LDL-c, HDL-c and TG, beta value, p-value for testing associations of LDL-c, HDL-c and TG with risk for CAD.

### Preparing *β* table for MVMR analysis

Once selection of SNPs was done using the above method, a *β* table is required for MVMR analysis. We followed the following steps to make a standard *β* table:

Step 1: retrieve *β*-values of SNPs associated with LDL-c, HDL-c, and/or TG by using SNPID from a merged data. For the convenience, these *β*- values were called LDL-, HDL-, and TG-betas.

Step 2: retrieve major allele of each SNP associated with LDL-c, HDL-c, and/or TG from the merged data. For the convenience, these alleles were called LDL-, HDL-, and TG-alleles.

Step 3: retrieve major allele (also called reference allele) of each SNP in CAD from the merged data, called CAD-allele.

Step 4: respectively compare LDL-, HDL-, and TG-alleles to CAD-allele. If they are different, then change sign of beta values of these lipid components (for example, - beta → beta or beta → -beta).

Step 5: put vectors CAD-, LDL-, HDL-, and TG-beta values corresponding to SNPs into a table.

### Performance of MR-PRESSO

We utilized MR-PRESSO^41^ to test for horizontal pleiotropic outliers on beta values and standard errors of SNPs selected in regression on each of the three lipid components and on CAD from datasets, datasets A and B. Before conducting the outlier test, the allele of a SNP associated with one lipid component was compared to one of this SNP associated with CAD and beta value of this lipid component was then changed with a positive or negative sign if alleles between them are different.

### R^2^ calculation

Alleles 1 and 2 of each SNP selected were used to construct genotypes, we calculated genotype and total variances V_g_(x) and V_t_(x) of beta values of SNP set associated with a lipid component (x). Then R^2^(x) = V_g_(x)/V_t_(x), where x = LDL-c, HDL-c or TG.

### Statistical analysis

We conducted multivariate MR (MVMR), simple median-based, weighted median-based, IVW, and MR-Egger^20^ methods to infer causality of lipid cholesterols on CAD using data from selected SNPs. We used R package, *MendelianRandomization*, to implement these single-variable MR analysis methods. MVMR statistically adjusts for multiple risk variables^15^. We used t-test in multivariate regression to test for association between each lipid component and the risk of CAD. MR-Egger is considered to account for unbalanced pleiotropy of a genetic instrument on a disease of study. Bowden *et al*.^20^ showed that the MR-Egger estimate is unaffected by net pleiotropic effects of the instrumental variables and that the presence of unbalanced pleiotropy can be inferred if the intercept term is not zero^15^. However, this approach does not adjust for pleiotropy of SNPs on multiple risk factors. We also performed correlation analysis and simulation to evaluate pleiotropy of SNPs on lipid components and disease.

### Packages, functions, and code for implementing MR analysis

Our SNP selection and MR analysis were conducted in R environment (https://www.r-project.org). Single-variable MR analysis was implemented by using R package, *MendelianRandomization*. MVMR and correlation analyses were carried out by performing R package, *GMRP*, in Bioconductor (https://bioconductor.org/packages/release/bioc/html/GMRP.html). R packages, *Forestplot*, *gride* and *remeta*, were used for making forest plots. MRPRESSO downloaded from CRAN was used to check horizontal pleiotropy of a SNP dataset.

## Supplementary information

Supplementary information.

Supplementary Datasets.
