# Supplementary material for: In-depth Mendelian randomization analysis of causal factors for coronary artery disease
Source: Sci Rep. 2020 Jun 8;10:9208. doi: 10.1038/s41598-020-66027-4 (PMC7280530; doi:10.1038/s41598-020-66027-4)
Supplement: Supplementary file 1 — Supplementary information. [file 41598_2020_66027_MOESM1_ESM.pdf]

# In-depth Mendelian randomization analysis of causal factors for coronary artery disease

Yuan-De Tan, Peng Xiao and Chittibabu Guda*

## Supplementary Notes:

### Note S1: $P_{cj}, P_{dj}, P_{vj}$ definitions

To correctly select SNPs from GWAS data or meta-GWAS data, we here defined $P_c$ and $P_d$ as proportion cutoffs in sample sizes of causal variables and of a disease of study, respectively, and defined $P_v$ as p-value cutoff in the causal data. We also defined

$$P_{cj} = \sum_{i=1}^{n} s_{ij} / max_{j=1}^{m}\left(\sum_{i=1}^{n} s_{ij}\right) \quad ,$$
$$P_{dj} = s_{dj} / max_{j=1}^{m}(s_{dj}) \quad ,$$
$$P_{vj} = \min_{j=1}^{m}(p_{ij})$$

where *n* is number of casual variables, *m*, number of SNPs in GWAS data, $s_{ij}$ , sample size for $SNP_j$ in causal variable *i*, $s_{dj}$, sample size for $SNP_j$ in the disease data and $p_{ij}$, p-value for t-testing for association of $SNP_j$ with casual variable *i* in regression analysis.

### Note S2: An algorithm for selecting SNPs with reducing linkage disequilibrium

Since most of GWAS data do not give linkage disequilibrium (LD) values between SNPs, we use the following algorithm to remove SNPs with strong LD. Suppose we have a GWAS dataset with G SNPs each having chromosome position (bp), major allele frequency, beta values, p-values, sample sizes(N) of LDL, HDL, TG, and CAD in GWAS analysis. To choose a SNP as a valid instrument variable, we first calculate $P_{vj}$, $P_{cj}$ and $P_{dj}$. The algorithm for choosing SNPs without strong LD is given as

Step 1: All SNPs meeting $P_{vj} < P_v$ are chosen from the GWAS data and sub-dataset A is generated with these SNPs from the GWAS data.

Step 2: All SNPs meeting $P_{cj} > P_c$ are chosen from the sub-dataset A and sub-dataset B is generated with these chosen SNPs from sub-dataset A.

Step 3: All SNPs meeting $P_{dj} > P_d$ are chosen from sub-dataset B and sub-dataset C is generated with these chosen SNPs from sub-dataset B.

Step 4: Order SNPs by chromosomes.

Step 5: Choose chromosome 1 and order SNPs by positions in hg19 from the smallest position to the largest one.

Step 6: Set DI =(largest position-smallest position)/AIL where DI is distance index and AIL is adjacent interval length.

Step 7: If $\text{DI} \leq 1$, then only one SNP at the smallest or largest position in this chromosome is chosen, otherwise, if distance between positions j+1 and j >AIL and distance between positions j+2 and j+1 >AIL, SNPs at positions j and j+1 are chosen where j =1, …, $N_i$ where N is the largest position on chromosome *i*.

Step 8: Repeat step 7 from j= position1 to the third largest position.

Step 9: Repeat step 5 through step 8 until chromosome 22.

Step 10: Use these SNP chosen to create sub-dataset D from sub-dataset C.

## Note S3: Mathematic models for balance between LDL-c and HDL-c to determine causal effect of TG on CAD

From the results which TG was not associated with the risk for CAD by using the 83 shared SNPs in the SNP338 dataset as instrumental variables (**Fig. 6a**), we can infer

that the 64 SNPs shared with HDL-c and TG (**Fig. 3d**) were associated with HDL-c but their genes mostly had strong negative pleiotropic effects on TG and although 35 shared SNPs with LDL-c and TG were associated with LDL-c, their genes mostly had strong positive pleiotropic effect on TG. In MVMR analysis, after adjusting for HDL-c and LDL-c, if pleiotropic effects of SNPs associated with HDL-c and with LDL-c were balanced, TG is not associated with CAD. These results can be explained by the following multivariate regression model:

$$\sum_{j=1}^{m} \beta_{CADj} = a + \beta_{TG} \sum_{j=1}^{m} \beta_{TGj} + \beta_{LDL} \sum_{j=1}^{m} \beta_{LDLj} + \beta_{HDL} \sum_{j=1}^{m} \beta_{HDLj} + \beta_{e} \sum_{j=1}^{m} e_j$$

where $a$ is intersection; $j$, $SNP_j$ ; $\beta_{xj}$, a regression coefficient of $SNP_j$ on a factor $x$ ($x$ = TG, HDL-c, LDL-c, or CAD); $\beta_x$, regression coefficient of risk factor $x$ on CAD; and $e_j$, random effect. When $m$ (number of SNPs selected) is larger, $\sum_{j}^{m} e_j \approx 0$. In testing for association of TG with risk for CAD using the shared SNPs as instrumental variables, if TG and HDL-c share $SNP_j$, both $|\beta_{TGj}|$ and $|\beta_{HDLj}|$ are larger, or if TG and LDL-c share $SNP_j$, both $|\beta_{TGj}|$ and $|\beta_{LDLj}|$ are larger. For the convenience, we replace $\beta_{TGj}$ with $\beta_{TGj_h}$ if $SNP_j$ is shared with HDL-c and TG or with $\beta_{TGj_l}$ if $SNP_j$ is shared with LDL-c and TG, the regression above can be changed as

$$\sum_{j}^{m} \beta_{CADj} = a + \beta_{TG} (\sum_{j_h}^{m_h} \beta_{TGj_h} + \sum_{j_l}^{m_l} \beta_{TGj_l}) + \beta_{LDL} \sum_{j}^{m} \beta_{LDLj} + \beta_{HDL} \sum_{j}^{m} \beta_{HDLj} + \beta_{e} \sum_{j}^{m} e_j$$

where $m_h + m_l = m$. In balance of pleiotropic effects between HDL-c and LDL-c, $\sum_{j_h}^{m_h} \beta_{TGj_h} + \sum_{j_l}^{m_l} \beta_{TGj_l} = 0$ and then $\beta_{TG} = 0$. This is case in the 83 shared SNPs in the SNP338 dataset (**Fig. 6a**). In the case of unbalance, if $\sum_{j_h}^{m_h} \beta_{TGj_h} + \sum_{j_l}^{m_l} \beta_{TGj_l} > 0$ and $\sum_{j}^{m} \beta_{CADj} > 0$, or $\sum_{j_h}^{m_h} \beta_{TGj_h} + \sum_{j_l}^{m_l} \beta_{TGj_l} < 0$ and $\sum_{j}^{m} \beta_{CADj} < 0$, then $\beta_{TG} > 0$ in statistics due to positive pleiotropic effect of SNPs associated with LDL-c, examples can

be found in the 99 (**Fig. 6c**) and 45 shared SNPs (**Fig. 6c**) in the SNP185 dataset or if $\sum_{j_h}^{m_h}\beta_{TGj_h}+\sum_{j_l}^{m_l}\beta_{TGj_l}<0$ but $\sum_{j}^{m}\beta_{CADj}>0$ or $\sum_{j_h}^{m_h}\beta_{TGj_h}+\sum_{j_l}^{m_l}\beta_{TGj_l}>0$ but $\sum_{j}^{m}\beta_{CADj}<0$ , then $\beta_{TG}<0$ in statistics due to negative pleiotropic effects of SNPs associated with HDL-c, like in the 116 shared SNPs in the SNP363 data (**Fig. 6b**).

Simulation breaks LD between SNPs j and k (no link between ($\beta_{xj}$, $\beta_{CADj}$) and ($\beta_{xk}$, $\beta_{CADk}$) ) and removes pleiotropy of SNPs between lipids $x$ and $y$ (no association between ($\beta_{xj}$, $\beta_{CADj}$) and ($\beta_{yj}$, $\beta_{CADj}$) ). Simulation tests for associations of LDL-c and HDL-c with risk for CAD in the SNP338 and SNP363 datasets in no noise and noise cases directly verified the conclusion above. For TG, the test SNPs consist of two groups: one group in which SNPs were associated with HDL-c and TG in the test data is similar to , as noted in the second regression model, $\beta_{TGj}$is referred to as $\beta_{TGj_h}$ for $SNP_j$, and the other group in which SNPs were associated with LDL-c and TG in the test data, the $\beta_{TGj}$ is referred to as $\beta_{TGj_l}$. If $\sum_{j=1}^{m_h}\beta_{TGj_h}$ and $\sum_{j=1}^{m_l}\beta_{TGj_l}$ are balanced to be zero, then $\beta_{TG}$ = 0. The case can be seen in simulations using SNP338 and SNP363 datasets as test datasets. For the SNP185 dataset of Do et al[3], many SNPs associated with HDL-c were not selected due to selection bias so that HDL-c was not associated with risk for CAD. On the other hand, as shown in Figure 3g, TG had 24 SNPs associated with HDL-c only but just 8 SNPs associated with LDL-c only. Table 2 also shows that in SNP185 dataset, TG was negatively correlated with HDL-c ($r$ = -0.30512, $p$ < 0.0001) but not correlated with LDL-c ( $r$ = 0.034, $p$ = 0.64). Therefore, in simulated data, $|\sum_{j=1}^{m_h}\beta_{TGj_h}|$ > $|\sum_{j=1}^{m_l}\beta_{TGj_l}|$, leading to $\beta_{TG}<0$ in statistics.

## **Supplementary Tables:**

Table S1. Data sources and information

| Data name | Risk factors or disease | Number of participates | SNP Number | Data sources or references |
|---|---|---|---|---|
| metabochip data (Mc) | LDL-c, HDL-c, TG | 188577 | 120165 | http://csg.sph.umich.edu/abecasis/public/lipids2013/ |
| Joint GWAS and metabochip data (jointGwasMc) | LDL-c, HDL-c, TG | 187167 | 2436375 | http://csg.sph.umich.edu/abecasis/public/lipids2013/ |
| CARDIoGRAMplusC4D | CAD | Cases: 63746 Controls: 130 681 | 2420360 | http://www.cardiogramplusc4d.org/downloads/ |
| Data of 185 SNPs of Do et al.(2013) | LDL-c, HDL-c, TG, and CAD | 188577 | 185 | http://www.nature.com/ng/journal/v45/n11/full/ng.2795.html |

CAD, coronary artery disease; CARIoGRAMplusC4D: Coronary Artery Disease Genome-wide Replication and Meta-analysis plus Coronary Artery Disease Genetics; GLGC, Global Lipids Genetic Consortium; HDL-c, high-density lipoprotein cholesterol; LDL-C; low-density lipoprotein cholesterol; TG: triglycerides; SNP: single nucleotide polymorphism.

Table S2. Selection schemes of SNPs

| Scheme A | | | | | Scheme B | | | | |
|---|---|---|---|---|---|---|---|---|---|
| AIL | $P_v$ | $P_c$ | $P_d$ | SNP# | AIL | $P_v$ | $P_c$ | $P_d$ | SNP# |
| 25000 | 5e-08 | 0.99 | 0.99 | 58 | 25000 | 5e-08 | 0.979 | 0.979 | 58 |
| 20000 | 5e-08 | 0.99 | 0.99 | 73 | 20000 | 5e-08 | 0.979 | 0.979 | 68 |
| 15000 | 5e-08 | 0.99 | 0.99 | 80 | 15000 | 5e-08 | 0.979 | 0.979 | 91 |
| 10000 | 5e-08 | 0.99 | 0.99 | 94 | 10000 | 5e-08 | 0.979 | 0.979 | 121 |
| 5000 | 5e-08 | 0.99 | 0.99 | 114 | 5000 | 5e-08 | 0.979 | 0.979 | 178 |
| 1000 | 5e-08 | 0.99 | 0.99 | 133 | 1000 | 5e-08 | 0.979 | 0.979 | 266 |
| | 5e-08 | 0.99 | 0.99 | 171 | | 5e-08 | 0.979 | 0.979 | 338 |
| | 5e-08 | 0.98 | 0.98 | 265 | | 5e-08 | 0.95 | 0.972 | 735 |
| | 5e-08 | 0.979 | 0.979 | 338 | | | | | |
| | 5e-08 | 0.972 | 0.972 | 582 | | | | | |
| | 5e-08 | 0.95 | 0.972 | 735 | | | | | |

$P_v$ is a given threshold for minimum p-value ($P_{vj}$) of SNPs associated with LDL-c, HDL-c and TG; $P_c$ , a given threshold for proportion ($P_{cj}$) of sum of sample size of SNP j over all causal variables to the max sum of sample sizes over all SNPs in data; $P_d$ , a given threshold for proportion ($P_{dj}$) of sample size of SNP j in disease to the max sample sizes over all sNPs (See **Supplementary Note S2**).

Table S3. Correlation analysis between LDL-c, HDL-c and TG

| from data | SNP # | | LDL-c | HDL-c | TG |
|---|---|---|---|---|---|
| Mc-lipid-CAD | 338 | LDL-c | 1 | 0.2114 | <0.0001 |
| | | HDL-c | -0.06668 | 1 | <0.0001 |
| | | TG | 0.420036 | -0.55972 | 1 |
| jointGwasMc-lipid-CAD | 363 | LDL-c | 1 | 0.090419 | <0.0001 |
| | | HDL-c | -0.08952 | 1 | <0.0001 |
| | | TG | 0.330894 | -0.6977 | 1 |
| data of white et al (2016) | 145 | LDL-c | 1 | 0.0719 | 0.5900 |
| | | HDL-c | -0.14991 | 1 | <0.0001 |
| | | TG | -0.04512 | -0.45708 | 1 |
| data of Do et al (2013) | 185 | LDL-c | 1 | 0.0526 | 0.6464 |
| | | HDL-c | -0.14312 | 1 | <0.0001 |
| | | TG | 0.034039 | -0.30512 | 1 |

Note: correlation coefficients are under diagonal lines and corresponding p-values are above diagonal lines.

Table S4. Results of MR-PRESSO analysis of dataset SNP338

| Exposure | MR Analysis | Causal Estimate | Sd | T-stat | P-value |
|---|---|---|---|---|---|
| ldl | Raw | 0.3559716 | 0.03506907 | 10.150586 | 2.764653e-21 |
| hdl | Raw | -0.3449137 | 0.03446413 | -10.007903 | 8.423297e-21 |
| tg | Raw | 0.1604794 | 0.03392713 | 4.730118 | 3.312579e-06 |
| ldl | Outlier-corrected | NA | NA | NA | NA |
| hdl | Outlier-corrected | NA | NA | NA | NA |
| tg | Outlier-corrected | NA | NA | NA | NA |

MR-PRESSO results`$`Global Test`$RSSobs: 550.0842
MR-PRESSO results`$`Global Test`$`Pvalue: <0.001
MR-PRESSO results`$`Distortion Test`$`Outliers Indice: No significant outliers
MR-PRESSO results`$`Distortion Test`$`Distortion Coefficient: NA
MR-PRESSO results`$`Distortion Test`$`Pvalue: NA

Table S5. Results of MR-PRESSO analyses of datasets SNP363 and SNP360

| data | Exposure | MR Analysis | Causal Estimate | Sd | T-stat | P-value |
|---|---|---|---|---|---|---|
| SNP338 | ldl | Raw | 0.42386 | 0.04038 | 10.496331 | 1.155620e-22 |
| | hdl | Raw | -0.38128 | 0.05090 | -7.490704 | 5.346223e-13 |
| | tg | Raw | 0.15585 | 0.05816 | 2.679619 | 7.709089e-03 |
| | ldl | Outlier-corrected | 0.51658 | 0.04571 | 11.301323 | 1.464482e-25 |
| | hdl | Outlier-corrected | 0.51658 | 0.04571 | 11.301323 | 1.464482e-25 |
| | tg | Outlier-corrected | 0.51658 | 0.04571 | 11.301323 | 1.464482e-25 |
| SNP360 (3 SNPs were excluded from SNP338) | ldl | Raw | 0.44025 | 0.03904 | 11.275608 | 1.978695e-25 |
| | hdl | Raw | -0.37571 | 0.04913 | -7.647783 | 1.916145e-13 |
| | tg | Raw | 0.15102 | 0.05609 | 2.692141 | 7.433924e-03 |
| | ldl | Outlier-corrected | NA | NA | NA | NA |
| | hdl | Outlier-corrected | NA | NA | NA | NA |
| | tg | Outlier-corrected | NA | NA | NA | NA |

MR-PRESSO results`$`Global Test`$RSSobs: 672.9603
MR-PRESSO results`$`Global Test`$`Pvalue: <0.001
MR-PRESSO results`$`Distortion Test`$`Outliers Indice: No significant outliers
MR-PRESSO results`$`Distortion Test`$`Distortion Coefficient: NA
MR-PRESSO results`$`Distortion Test`$`Pvalue: NA

Table S6. $R^2$ of SNP datasets

| | | LDL-c | HDL-c | TG |
|---|---|---|---|---|
| **SNP338** | **total** | 0.067658 | 0.020118 | 0.010047 |
| | **shared** | 0.118296 | 0.372035 | 0.135062 |
| | **unique** | 0.158198 | 0.071825 | 0.170607 |
| **SNP363** | **total** | 0.058397 | 0.004520 | 0.030580 |
| | **shared** | 0.036203 | 0.116992 | 0.082518 |
| | **unique** | 0.114813 | 0.047842 | 0.080932 |
| **SNP173(1)** | | 0.069171 | 0.021500 | 0.037258 |
| **SNP173(2)** | | 0.065374 | 0.018952 | 0.033582 |
| **SNP185** | | 0.015385 | 0.162740 | 0.028591 |
| **SNP145** | | 0.038207 | 0.328188 | 0.038345 |

Table S7. Results [a] of simulation MVMR analysis of lipid risk for coronary artery disease using SNP338 data as test data and Mc-lipid-CAD data as mother data

| | | SNP number found | | | | | LDL-c | | HLD-c | | TG | |
|---|---|---|---|---|---|---|---|---|---|---|---|---|
| Pc | Pd | Total | LDL-c | HDL-c | TG | FDR | beta | p-value | beta | p-value | beta | p-value |
| **null p-value is distributed from 1e-07 to 1** | | | | | | | | | | | | |
| 0.9 | 0.9 | 394 | 101 | 148 | 148 | 0.0 | 0.430 | 3.83E-14 | -0.392 | 2.40E-16 | -0.034 | 0.35993 |
| 0.9 | 0.98 | 319 | 76 | 124 | 122 | 0.0 | 0.462 | 1.76E-11 | -0.394 | 2.98E-13 | -0.031 | 0.40845 |
| 0.9 | 0.99 | 212 | 50 | 84 | 79 | 0.0 | 0.451 | 5.15E-08 | -0.401 | 1.10E-09 | 0.020 | 0.58740 |
| 0.95 | 0.9 | 339 | 85 | 128 | 129 | 0.0 | 0.426 | 6.93E-12 | -0.382 | 4.55E-12 | -0.026 | 0.43788 |
| 0.95 | 0.98 | 278 | 64 | 109 | 108 | 0.0 | 0.455 | 1.95E-09 | -0.389 | 4.81E-10 | -0.026 | 0.46385 |
| 0.95 | 0.99 | 183 | 42 | 74 | 69 | 0.0 | 0.439 | 7.63E-06 | -0.397 | 9.90E-08 | 0.023 | 0.47068 |
| 0.97 | 0.9 | 292 | 73 | 109 | 112 | 0.0 | 0.435 | 9.41E-10 | -0.379 | 1.46E-09 | -0.028 | 0.43603 |
| 0.97 | 0.98 | 241 | 56 | 94 | 94 | 0.0 | 0.464 | 4.89E-08 | -0.381 | 2.41E-08 | -0.027 | 0.49519 |
| 0.97 | 0.99 | 160 | 37 | 65 | 60 | 0.0 | 0.439 | 5.01E-05 | -0.396 | 4.42E-06 | 0.011 | 0.37889 |
| 0.98 | 0.9 | 239 | 60 | 88 | 92 | 0.0 | 0.445 | 1.32E-08 | -0.380 | 3.76E-08 | -0.036 | 0.31616 |
| 0.98 | 0.98 | 197 | 46 | 75 | 77 | 0.0 | 0.478 | 6.62E-07 | -0.384 | 5.78E-07 | -0.034 | 0.31816 |
| 0.98 | 0.99 | 129 | 30 | 51 | 49 | 0.0 | 0.443 | 0.00896 | -0.400 | 5.42E-05 | 0.005 | 0.16253 |
| 0.99 | 0.9 | 219 | 56 | 81 | 85 | 0.0 | 0.451 | 7.97E-08 | -0.369 | 1.31E-06 | -0.039 | 0.29322 |
| 0.99 | 0.98 | 181 | 43 | 69 | 71 | 0.0 | 0.482 | 3.34E-06 | -0.372 | 2.50E-05 | -0.039 | 0.35592 |
| 0.99 | 0.99 | 119 | 28 | 47 | 45 | 0.0 | 0.444 | 0.01441 | -0.393 | 0.00015 | -0.007 | 0.19769 |
| Min | | 119 | 28 | 47 | 45 | 0.0 | 0.426 | 3.83E-14 | -0.401 | 2.4E-16 | -0.039 | 0.16253 |
| Max | | 394 | 101 | 148 | 148 | 0.0 | 0.482 | 0.01441 | -0.369 | 0.00015 | 0.023 | 0.5874 |
| mean | | 233 | 56 | 90 | 90 | 0.0 | 0.449 | 0.00156 | -0.387 | 1.6E-05 | -0.018 | 0.37879 |
| **null p-value is distributed from 1e-12 to 1** | | | | | | | | | | | | |
| 0.9 | 0.9 | 456 | 102 | 149 | 147 | 0.136 | 0.224 | 0.00692 | -0.199 | 0.00098 | -0.0303 | 0.54951 |
| 0.9 | 0.95 | 433 | 102 | 149 | 147 | 0.088 | 0.227 | 0.00622 | -0.199 | 0.00099 | -0.0283 | 0.53214 |
| 0.9 | 0.97 | 416 | 102 | 149 | 147 | 0.053 | 0.229 | 0.00662 | -0.202 | 0.00078 | -0.0296 | 0.50381 |
| 0.9 | 0.98 | 328 | 77 | 125 | 120 | 0.024 | 0.237 | 0.00717 | -0.191 | 0.00580 | -0.0232 | 0.48904 |
| 0.95 | 0.9 | 390 | 87 | 129 | 128 | 0.126 | 0.227 | 0.00526 | -0.203 | 0.00183 | -0.0492 | 0.40744 |
| 0.95 | 0.95 | 371 | 87 | 129 | 128 | 0.081 | 0.230 | 0.00402 | -0.204 | 0.00208 | -0.0473 | 0.40318 |
| 0.95 | 0.97 | 358 | 87 | 129 | 128 | 0.047 | 0.231 | 0.00421 | -0.205 | 0.00202 | -0.048 | 0.39936 |
| 0.95 | 0.98 | 284 | 66 | 108 | 105 | 0.021 | 0.236 | 0.00899 | -0.196 | 0.00905 | -0.0409 | 0.44766 |
| 0.97 | 0.9 | 339 | 78 | 113 | 112 | 0.115 | 0.227 | 0.00686 | -0.203 | 0.00591 | -0.0606 | 0.38646 |
| 0.97 | 0.95 | 324 | 78 | 113 | 112 | 0.074 | 0.230 | 0.00552 | -0.204 | 0.00508 | -0.0586 | 0.40456 |
| 0.97 | 0.97 | 314 | 78 | 113 | 112 | 0.045 | 0.230 | 0.00608 | -0.206 | 0.00581 | -0.0597 | 0.39458 |
| 0.97 | 0.98 | 250 | 60 | 95 | 93 | 0.02 | 0.231 | 0.02614 | -0.197 | 0.01790 | -0.0571 | 0.41751 |
| 0.98 | 0.9 | 280 | 66 | 94 | 92 | 0.107 | 0.217 | 0.02392 | -0.213 | 0.01216 | -0.036 | 0.60610 |
| 0.98 | 0.95 | 268 | 66 | 94 | 92 | 0.071 | 0.220 | 0.02001 | -0.213 | 0.01066 | -0.034 | 0.57882 |
| 0.98 | 0.97 | 260 | 66 | 94 | 92 | 0.038 | 0.220 | 0.02133 | -0.216 | 0.01075 | -0.0354 | 0.56723 |
| 0.98 | 0.98 | 208 | 51 | 78 | 77 | 0.019 | 0.221 | 0.03510 | -0.208 | 0.04489 | -0.0323 | 0.53354 |
| 0.99 | 0.9 | 255 | 60 | 86 | 85 | 0.102 | 0.215 | 0.01770 | -0.223 | 0.00903 | -0.0351 | 0.50215 |
| 0.99 | 0.95 | 245 | 60 | 86 | 85 | 0.065 | 0.217 | 0.01486 | -0.222 | 0.01003 | -0.033 | 0.49443 |
| 0.99 | 0.97 | 238 | 60 | 86 | 85 | 0.038 | 0.216 | 0.01641 | -0.226 | 0.00877 | -0.0348 | 0.49136 |

| | | | | | | | | | | | | |
|---|---|---|---|---|---|---|---|---|---|---|---|---|
| 0.99 | 0.98 | 191 | 47 | 72 | 70 | 0.021 | 0.218 | 0.03019 | -0.218 | 0.03649 | -0.0323 | 0.50339 |
| Min | | 191 | 47 | 72 | 70 | 0.019 | 0.215 | 0.00402 | -0.226 | 0.00078 | -0.061 | 0.38646 |
| Max | | 456 | 102 | 149 | 147 | 0.136 | 0.237 | 0.03510 | -0.191 | 0.04489 | -0.023 | 0.60610 |
| mean | | 310 | 74 | 110 | 108 | 0.064 | 0.225 | 0.01367 | -0.207 | 0.01005 | -0.040 | 0.48061 |

[a] Results in the table are averaged over 10 repeat simulations. $P_c$ is a given criterion for proportion of sum of sample sizes of a SNP over all given causal variables to the largest sum of sample sizes over all SNPs in a simulated dataset and $P_d$ , given criterion for proportion of sample size of a SNP in a disease to the largest sample size over all SNPs in the disease in a simulated dataset.

Table S8. Results [a] of simulation MVMR analysis of lipid risk for coronary artery disease using dataset SNP185 of Do et al as test data and Mc-lipid-CAD data as mother data

| | | Number of SNPs found | | | | | LDL-c | | HDL-c | | TG | |
|---|---|---|---|---|---|---|---|---|---|---|---|---|
| | | total | LDL | HDL | TG | FDR | beta | p-value | beta | p-value | beta | p-value |
| Pc | Pd | | | | | **null p-value is distributed from 1e-07 to 1** | | | | | | |
| | | 238 | 82 | 96 | 60 | 0 | 0.391 | 2.98E-15 | -0.129 | 0.01489 | -0.298 | 0.00018 |
| 0 | 0.7 | 223 | 77 | 90 | 57 | 0 | 0.391 | 2.42E-14 | -0.126 | 0.02124 | -0.328 | 0.00015 |
| 0 | 0.9 | 155 | 53 | 63 | 39 | 0 | 0.392 | 3.75E-08 | -0.113 | 0.09856 | -0.308 | 0.01696 |
| 0 | 0.915 | 143 | 49 | 58 | 36 | 0 | 0.385 | 1.52E-07 | -0.114 | 0.09327 | -0.314 | 0.01541 |
| 0 | 0.93 | 128 | 45 | 51 | 33 | 0 | 0.365 | 0.001954 | -0.109 | 0.12561 | -0.282 | 0.0999 |
| 0.7 | 0 | 237 | 82 | 96 | 60 | 0 | 0.391 | 3.32E-15 | -0.129 | 0.01495 | -0.298 | 0.00018 |
| 0.7 | 0.7 | 222 | 77 | 90 | 56 | 0 | 0.391 | 3.00E-14 | -0.125 | 0.02125 | -0.329 | 0.00016 |
| 0.7 | 0.9 | 154 | 53 | 63 | 39 | 0 | 0.391 | 5.97E-08 | -0.113 | 0.09825 | -0.308 | 0.01705 |
| 0.7 | 0.915 | 142 | 49 | 58 | 36 | 0 | 0.384 | 2.06E-07 | -0.113 | 0.09306 | -0.315 | 0.01553 |
| 0.7 | 0.93 | 128 | 45 | 51 | 33 | 0 | 0.365 | 0.001954 | -0.109 | 0.12564 | -0.283 | 0.09989 |
| 0.9 | 0 | 204 | 71 | 83 | 52 | 0 | 0.394 | 2.71E-12 | -0.131 | 0.03048 | -0.305 | 0.00136 |
| 0.9 | 0.7 | 191 | 66 | 77 | 49 | 0 | 0.393 | 1.04E-10 | -0.126 | 0.04899 | -0.332 | 0.00108 |
| 0.9 | 0.9 | 132 | 46 | 54 | 33 | 0 | 0.391 | 2.38E-07 | -0.100 | 0.18609 | -0.294 | 0.09471 |
| 0.9 | 0.915 | 122 | 43 | 50 | 30 | 0 | 0.386 | 6.31E-07 | -0.100 | 0.18839 | -0.317 | 0.07237 |
| 0.9 | 0.93 | 109 | 39 | 44 | 27 | 0 | 0.366 | 0.003088 | -0.094 | 0.20085 | -0.285 | 0.14139 |
| 0.915 | 0 | 196 | 68 | 79 | 50 | 0 | 0.402 | 7.55E-10 | -0.126 | 0.03853 | -0.297 | 0.00314 |
| 0.915 | 0.7 | 183 | 64 | 74 | 47 | 0 | 0.402 | 1.40E-09 | -0.121 | 0.05929 | -0.322 | 0.00327 |
| 0.915 | 0.9 | 126 | 44 | 51 | 32 | 0 | 0.4 | 2.70E-07 | -0.094 | 0.18848 | -0.28 | 0.11201 |
| 0.915 | 0.915 | 116 | 41 | 47 | 29 | 0 | 0.395 | 7.06E-07 | -0.093 | 0.20179 | -0.302 | 0.09169 |
| 0.915 | 0.93 | 104 | 37 | 42 | 26 | 0 | 0.374 | 0.006387 | -0.09 | 0.20142 | -0.264 | 0.16553 |
| 0.93 | 0 | 187 | 65 | 75 | 47 | 0 | 0.399 | 7.33E-10 | -0.135 | 0.03407 | -0.301 | 0.00282 |
| 0.93 | 0.7 | 175 | 62 | 70 | 44 | 0 | 0.4 | 1.97E-09 | -0.130 | 0.05223 | -0.316 | 0.00349 |
| 0.93 | 0.9 | 120 | 43 | 49 | 30 | 0 | 0.398 | 4.77E-07 | -0.097 | 0.19502 | -0.281 | 0.13618 |
| 0.93 | 0.915 | 111 | 40 | 46 | 27 | 0 | 0.392 | 1.04E-06 | -0.097 | 0.20669 | -0.306 | 0.1214 |
| 0.93 | 0.93 | 99 | 36 | 41 | 24 | 0 | 0.37 | 0.008527 | -0.093 | 0.19999 | -0.264 | 0.19548 |

| | | | | | | | | | | | | |
|---|---|---|---|---|---|---|---|---|---|---|---|---|
| Min | | 99 | 36 | 41 | 24 | 0 | 0.365 | 3.32E-15 | -0.135 | 0.01495 | -0.332 | 0.00016 |
| Max | | 237 | 82 | 96 | 60 | 0 | 0.402 | 0.00853 | -0.090 | 0.20669 | -0.264 | 0.19548 |
| mean | | 151 | 53 | 61 | 38 | 0 | 0.388 | 0.00099 | -0.111 | 0.118379 | -0.299 | 0.06336 |
| **null p-value is distributed from 1e-12 to 1** | | | | | | | | | | | | |
| 0 | 0 | 352 | 82 | 96 | 61 | 0.324 | 0.384 | 2.79E-14 | -0.1322 | 0.00778 | -0.246 | 0.00170 |
| 0 | 0.7 | 329 | 77 | 90 | 57 | 0.322 | 0.382 | 5.63E-13 | -0.1241 | 0.01499 | -0.266 | 0.01155 |
| 0 | 0.9 | 224 | 53 | 62 | 37 | 0.317 | 0.394 | 7.45E-07 | -0.1287 | 0.09683 | -0.242 | 0.17330 |
| 0 | 0.915 | 206 | 49 | 58 | 34 | 0.316 | 0.401 | 3.55E-06 | -0.1252 | 0.11716 | -0.254 | 0.15864 |
| 0 | 0.93 | 185 | 44 | 53 | 31 | 0.314 | 0.411 | 2.16E-06 | -0.1353 | 0.12906 | -0.264 | 0.14943 |
| 0.7 | 0 | 350 | 82 | 95 | 60 | 0.323 | 0.384 | 2.77E-14 | -0.1299 | 0.01172 | -0.245 | 0.00183 |
| 0.7 | 0.7 | 327 | 76 | 89 | 57 | 0.324 | 0.383 | 5.53E-13 | -0.1218 | 0.02256 | -0.265 | 0.01201 |
| 0.7 | 0.9 | 222 | 53 | 62 | 37 | 0.315 | 0.396 | 6.97E-07 | -0.1245 | 0.12675 | -0.240 | 0.18122 |
| 0.7 | 0.915 | 204 | 49 | 58 | 34 | 0.314 | 0.402 | 3.37E-06 | -0.1208 | 0.14668 | -0.253 | 0.16470 |
| 0.7 | 0.93 | 184 | 44 | 52 | 31 | 0.31 | 0.412 | 2.01E-06 | -0.131 | 0.15974 | -0.261 | 0.14281 |
| 0.9 | 0 | 301 | 70 | 83 | 51 | 0.326 | 0.387 | 3.34E-11 | -0.1372 | 0.02990 | -0.238 | 0.02082 |
| 0.9 | 0.7 | 281 | 65 | 77 | 48 | 0.324 | 0.384 | 1.98E-10 | -0.1285 | 0.04534 | -0.266 | 0.01822 |
| 0.9 | 0.9 | 190 | 45 | 54 | 31 | 0.316 | 0.403 | 2.08E-06 | -0.1254 | 0.14790 | -0.263 | 0.14550 |
| 0.9 | 0.915 | 176 | 41 | 50 | 29 | 0.313 | 0.414 | 2.44E-05 | -0.122 | 0.17048 | -0.263 | 0.13492 |
| 0.9 | 0.93 | 158 | 37 | 45 | 26 | 0.31 | 0.422 | 2.43E-05 | -0.1344 | 0.18013 | -0.256 | 0.14131 |
| 0.915 | 0 | 292 | 68 | 80 | 49 | 0.325 | 0.393 | 1.80E-10 | -0.1325 | 0.03306 | -0.238 | 0.02454 |
| 0.915 | 0.7 | 272 | 64 | 75 | 46 | 0.327 | 0.392 | 5.14E-09 | -0.123 | 0.05150 | -0.265 | 0.02427 |
| 0.915 | 0.9 | 185 | 44 | 52 | 30 | 0.319 | 0.411 | 5.34E-06 | -0.1183 | 0.15294 | -0.257 | 0.19116 |
| 0.915 | 0.915 | 170 | 41 | 48 | 28 | 0.318 | 0.413 | 3.70E-05 | -0.1141 | 0.17602 | -0.257 | 0.17377 |
| 0.915 | 0.93 | 153 | 37 | 44 | 25 | 0.314 | 0.422 | 4.30E-05 | -0.1345 | 0.18612 | -0.248 | 0.17767 |
| 0.93 | 0 | 278 | 64 | 76 | 47 | 0.324 | 0.389 | 4.53E-10 | -0.1319 | 0.03234 | -0.210 | 0.12062 |
| 0.93 | 0.7 | 260 | 60 | 71 | 44 | 0.323 | 0.388 | 5.63E-08 | -0.1234 | 0.04670 | -0.236 | 0.10573 |
| 0.93 | 0.9 | 176 | 41 | 49 | 29 | 0.318 | 0.411 | 2.21E-05 | -0.1228 | 0.14640 | -0.204 | 0.22578 |
| 0.93 | 0.915 | 162 | 38 | 46 | 27 | 0.315 | 0.415 | 3.92E-05 | -0.117 | 0.17288 | -0.198 | 0.27816 |
| 0.93 | 0.93 | 146 | 34 | 42 | 24 | 0.315 | 0.421 | 0.000324 | -0.1374 | 0.17824 | -0.207 | 0.15736 |
| Min | | 146 | 34 | 42 | 24 | 0.31 | 0.383 | 2.77E-14 | -0.1374 | 0.01172 | -0.266 | 0.00183 |
| Max | | 350 | 82 | 95 | 60 | 0.327 | 0.422 | 0.000324 | -0.1141 | 0.18612 | -0.198 | 0.27816 |
| mean | | 222 | 52 | 62 | 37 | 0.318 | 0.402 | 2.42E-05 | -0.1268 | 0.11198 | -0.245 | 0.12502 |

[a] Results in table are averaged over 10 repeat simulations. $P_c$ is a given criterion for proportion of sum of sample sizes of a SNP over all given causal variables to the largest sum of sample sizes over all SNPs in a simulated dataset and $P_d$, given criterion for proportion of sample size of a SNP in a disease to the largest sample size over all SNPs in the disease in a simulated dataset.

Table S9. Results [a] of simulation MVMR analysis of lipid risk for coronary artery disease using SNP363 data as test data and Mc-lipid-CAD data as mother data

| | | Number of SNPs found | | | | | LDL-c | | HDL-c | | TG | |
|---|---|---|---|---|---|---|---|---|---|---|---|---|
| Pc | Pd | total | LDL-c | HDL-c | TG | FDR | beta | pvalue | beta | p-value | beta | p-value |
| **null p-value is distributed from 1e-12 to 1** | | | | | | | | | | | | |
| 0 | 0 | 612 | 151 | 239 | 109 | 0.19 | 0.2705 | 2.85E-06 | -0.2144 | 2.50E-03 | -0.0661 | 0.254342 |
| 0 | 0.7 | 601 | 151 | 239 | 109 | 0.175 | 0.2717 | 2.65E-06 | -0.2167 | 2.74E-03 | -0.0668 | 0.263725 |
| 0 | 0.9 | 566 | 151 | 239 | 109 | 0.124 | 0.2743 | 9.56E-07 | -0.223 | 2.15E-03 | -0.0679 | 0.267804 |
| 0 | 0.93 | 554 | 151 | 239 | 109 | 0.105 | 0.2741 | 1.38E-06 | -0.2248 | 2.18E-03 | -0.0708 | 0.273109 |
| 0.5 | 0 | 604 | 151 | 239 | 109 | 0.179 | 0.2706 | 2.55E-06 | -0.2158 | 1.99E-03 | -0.0674 | 0.244669 |
| 0.5 | 0.7 | 594 | 151 | 239 | 109 | 0.165 | 0.2725 | 1.93E-06 | -0.2176 | 2.76E-03 | -0.068 | 0.245366 |
| 0.5 | 0.9 | 562 | 151 | 239 | 109 | 0.117 | 0.2748 | 8.46E-07 | -0.2242 | 2.12E-03 | -0.0694 | 0.262287 |
| 0.5 | 0.93 | 551 | 151 | 239 | 109 | 0.1 | 0.2744 | 1.15E-06 | -0.225 | 2.11E-03 | -0.0716 | 0.264578 |
| 0.7 | 0 | 478 | 145 | 232 | 105 | 0 | 0.2839 | 1.05E-06 | -0.2316 | 1.81E-03 | -0.0750 | 0.252553 |
| 0.7 | 0.7 | 478 | 145 | 232 | 105 | 0 | 0.2839 | 1.05E-06 | -0.2316 | 1.81E-03 | -0.0750 | 0.252553 |
| 0.7 | 0.9 | 478 | 145 | 232 | 105 | 0 | 0.2839 | 1.05E-06 | -0.2316 | 1.81E-03 | -0.0750 | 0.252553 |
| 0.7 | 0.93 | 478 | 145 | 232 | 105 | 0 | 0.2839 | 1.05E-06 | -0.2316 | 1.81E-03 | -0.0750 | 0.252553 |
| min | | 478 | 145 | 232 | 105 | 0 | 0.2705 | 8.46E-07 | -0.2316 | 0.00181 | -0.0750 | 0.244669 |
| max | | 612 | 151 | 239 | 109 | 0.19 | 0.2839 | 2.85E-06 | -0.2144 | 0.00276 | -0.0661 | 0.273109 |
| mean | | 546 | 149 | 237 | 108 | 0.096 | 0.2765 | 1.543E-06 | -0.2240 | 0.00215 | -0.0707 | 0.2571743 |

[a] Results in table are averaged over 10 repeat simulations. $P_c$ is a given criterion for proportion of sum of sample sizes of a SNP over all given causal variables to the largest sum of sample sizes over all SNPs in a simulated dataset and $P_d$, given criterion for proportion of sample size of a SNP in a disease to the largest sample size over all SNPs in the disease in a simulated dataset.

**<u>Supplementary Figures</u>**:

**Figure S1. Scatter error-bar plots of lipoprotein-cholesterols versus coronary artery disease in the data of unique SNPs**

Panels a, b, and c are respectively scatter error-bar plots of genetic associations of SNPs with LDL-c, HDL-c, and TG versus those with risk for CAD based on data of 255 unique SNPs in the data of 338 SNPs from Mc-lipid-CAD data and 247 unique SNPS in the data of 363 SNPs from jointGwasMc-lipid-CAD. Red solid line is a general liner regression line and green dash line is an MR-Egger regression line.

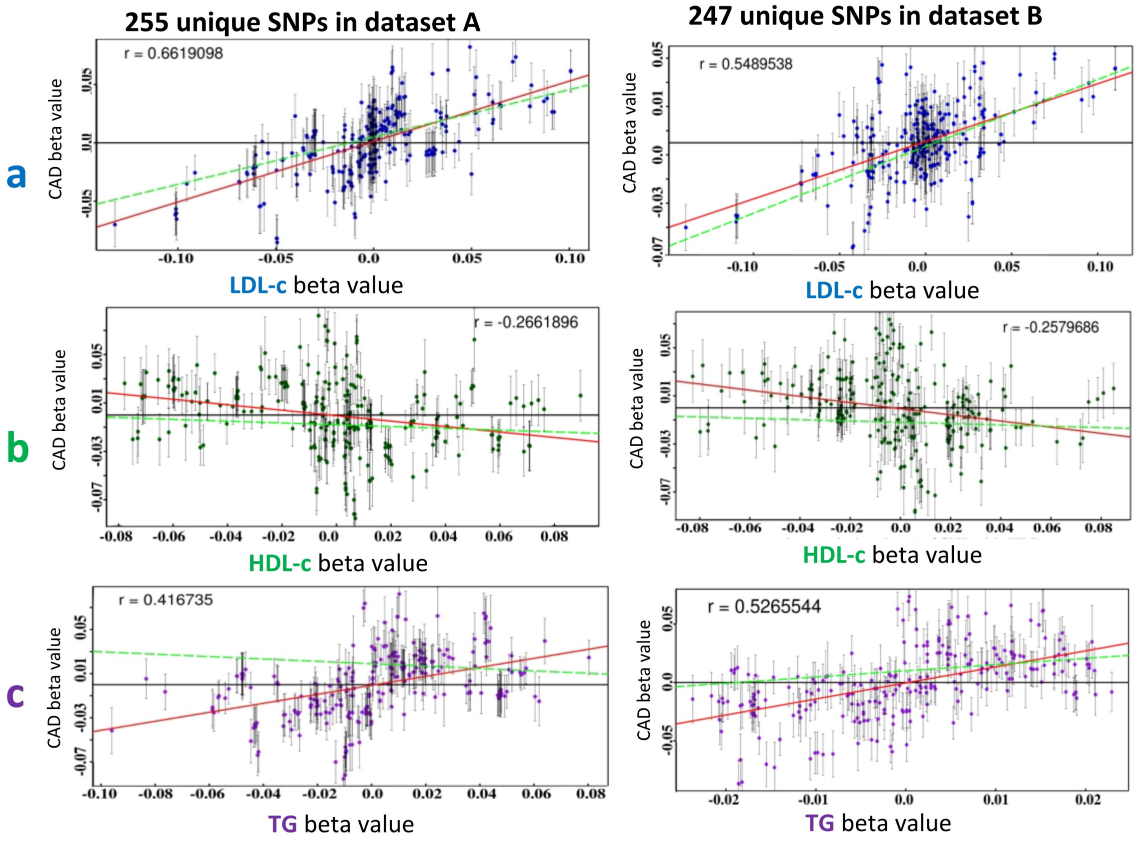

**Figure S2. Scatter error-bar plots of lipoprotein-cholesterols versus coronary artery disease in the data of shared SNPs**

Panels a, b, and c are respectively scatter errorbarplots of genetic associations of SNPs with LDL-c, HDL-c, and TG versus those with risk for CAD based on the data of 83 unique SNPs in the data of 338 SNPs from Mc-lipid-CAD data and 116 shared SNPS in the data of 363 SNPs from jointGwasMc-lipid-CAD. Red solid line is a general liner regression line and green dash line is an MR-Egger regression line.

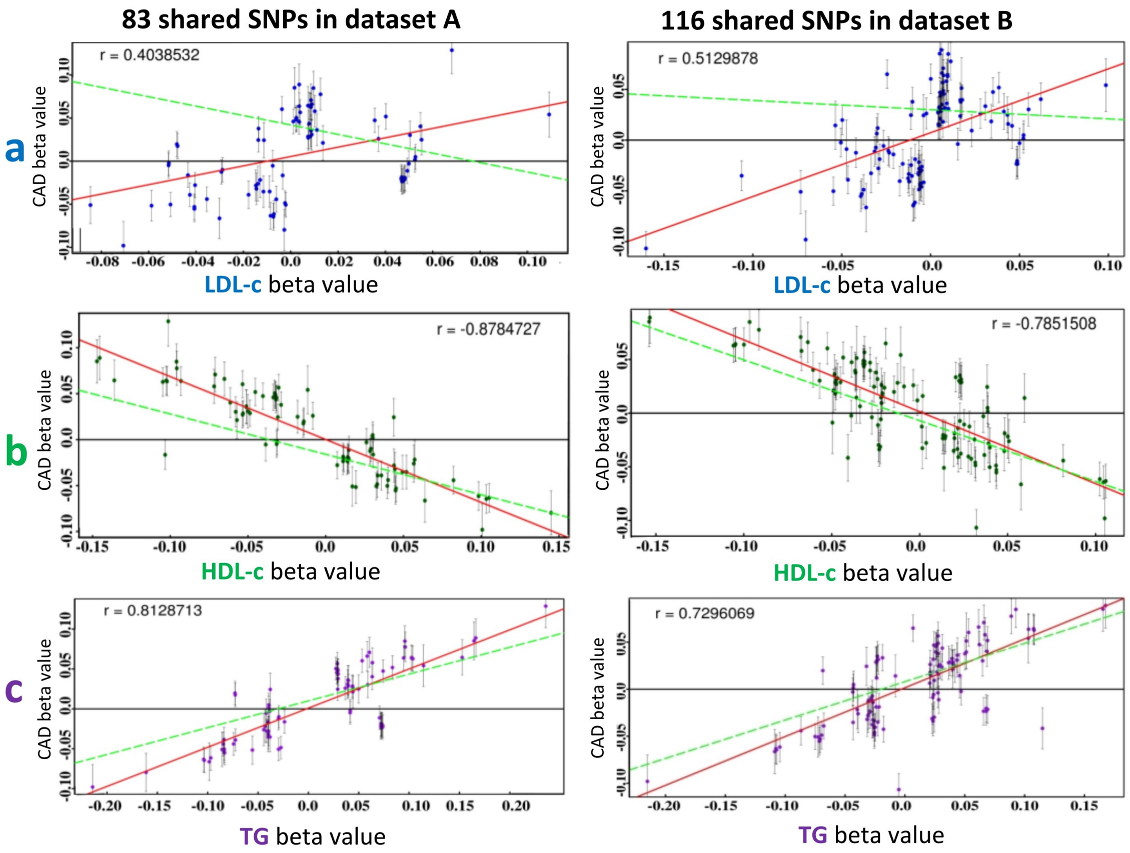

**Supplementary data (in Excel file with tabs for the following):**

1. Dataset A: 338SNPs_lipid_CAD
2. Dataset B: 363SNPs_lipid_CAD
3. Dataset D1: 173 SNPs_lipid_CAD_Mc
4. Dataset D2: 173SNPs_lpid_CAD_jontGwasMC

**Supplementary R code** for implementing SNP selection, MVMR analysis, drawing forestplot, errorbarplot, and large-scale simulation MVMR analysis will be uploaded on Github.
